# Zebrafish Mbd5 binds to RNA m^5^C and regulates histone deubiquitylation and gene expression in development metabolism and behavior

**DOI:** 10.1093/nar/gkae093

**Published:** 2024-02-15

**Authors:** Jianhua Guo, Zhongyu Zou, Xiaoyang Dou, Xiang Zhao, Yimin Wang, Liqiang Wei, Yan Pi, Yi Wang, Chuan He, Su Guo

**Affiliations:** State Key Laboratory of Genetic Engineering, National Demonstration Center for Experimental Biology Education, School of Life Sciences, Fudan University, Shanghai, China; Department of Chemistry and Howard Hughes Medical Institute, The University of Chicago, Chicago, IL 60637, USA; Department of Chemistry and Howard Hughes Medical Institute, The University of Chicago, Chicago, IL 60637, USA; Department of Bioengineering and Therapeutic Sciences, Programs in Human Genetics and Biological Sciences, University of California, San Francisco, CA 94143, USA; Department of Neurology, Children's Hospital of Fudan University, National Children's Medical Center, No. 399, Wanyuan Road, Minhang District, Shanghai, China; State Key Laboratory of Genetic Engineering, National Demonstration Center for Experimental Biology Education, School of Life Sciences, Fudan University, Shanghai, China; Department of Bioengineering and Therapeutic Sciences, Programs in Human Genetics and Biological Sciences, University of California, San Francisco, CA 94143, USA; State Key Laboratory of Genetic Engineering, National Demonstration Center for Experimental Biology Education, School of Life Sciences, Fudan University, Shanghai, China; Department of Bioengineering and Therapeutic Sciences, Programs in Human Genetics and Biological Sciences, University of California, San Francisco, CA 94143, USA; Department of Neurology, Children's Hospital of Fudan University, National Children's Medical Center, No. 399, Wanyuan Road, Minhang District, Shanghai, China; Department of Chemistry and Howard Hughes Medical Institute, The University of Chicago, Chicago, IL 60637, USA; Department of Bioengineering and Therapeutic Sciences, Programs in Human Genetics and Biological Sciences, University of California, San Francisco, CA 94143, USA

## Abstract

Complex biological processes are regulated by both genetic and epigenetic programs. One class of epigenetic modifications is methylation. Evolutionarily conserved methyl-CpG-binding domain (MBD)-containing proteins are known as readers of DNA methylation. MBD5 is linked to multiple human diseases but its mechanism of action remains unclear. Here we report that the zebrafish Mbd5 does not bind to methylated DNA; but rather, it directly binds to 5-methylcytosine (m^5^C)-modified mRNAs and regulates embryonic development, erythrocyte differentiation, iron metabolism, and behavior. We further show that Mbd5 facilitates removal of the monoubiquitin mark at histone H2A-K119 through an interaction with the Polycomb repressive deubiquitinase (PR-DUB) complex *in vivo*. The direct target genes of Mbd5 are enriched with both RNA m^5^C and H2A-K119 ubiquitylation signals. Together, we propose that zebrafish MBD5 is an RNA m^5^C reader that potentially links RNA methylation to histone modification and in turn transcription regulation *in vivo*.

## Graphical Abstract

**Graphical Abstract:**
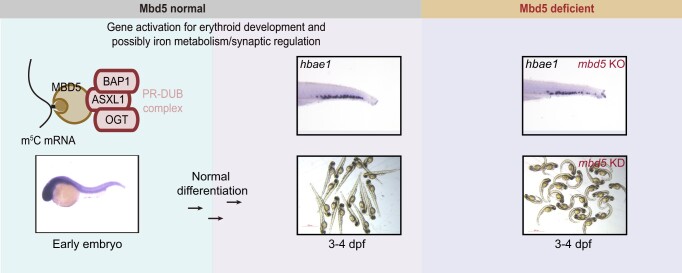

## Introduction

Individual cells in a multicellular organism contain an essentially identical genome that is epigenetically modified to enable differential gene expression and in turn distinct cellular identity. Methylation is one class of epigenetic modification, which can be recognized by methyl-CpG-binding domain (MBD)-containing proteins including MeCP2 and MBD1-6 (1,2). MeCP2, mutations in which cause the Rett syndrome (3), is the first MBD-containing protein discovered (4) that binds to 5-methylcytosine (5mC) in the CpG DNA (5). MBDs 1–4 also bind to methylated CpG DNA (6).

MBD5 in humans is mutated in multiple diseases ranging from autism spectrum disorders (ASD), epilepsy, to dementia (7–9). Previous studies employing rodent models describe a role of MBD5 in growth, glucose homeostasis, iron metabolism and behavior (10–12). A biochemical study in cultured cells uncovers an interaction of human MBD5 with the Polycomb repressive deubiquitinase (PR-DUB) complex (13). Unlike other MBD proteins, human MBD5 is unable to bind methylated DNA *in vitro* (14), but the plant MBD5 is reported to bind to methylated DNA (15). This leaves open the question as to what vertebrate MBD5 binds and what its mechanism of action is.

Here we use the zebrafish model to show that the zebrafish *mbd5* is a maternally expressed gene critical for embryonic development, iron metabolism and behavior. Mbd5 does not bind to methylated DNA. *In vivo* CLIP-seq uncovers that Mbd5 directly binds to m^5^C-modified mRNAs involved in erythrocyte differentiation, iron metabolism, and synaptic development. *In vivo* co-immunoprecipitation (IP) followed by mass spectrometry uncovers that Mbd5 interacts with a single protein complex, namely, the histone remodeling complex PR-DUB. Through this interaction, Mbd5 facilitates the removal of monoubiquitin, a repressive chromatin mark at the residue Lysine 119 of histone H2A (H2A-K119ub1). Mbd5’s direct target loci show enrichment for both RNA m^5^C modification and H2A-K119ub1 signals. Together, our findings establish that Mbd5 is an RNA m^5^C reader and a regulator of gene expression through promoting histone deubiquitylation *in vivo*.

## Materials and methods

### Key resources table

See Table 1.

**Table 1.** Key resources table

Reagent or resource	Source	Identifier
Antibodies		
Histone H3 Rabbit mAb	Cell Signaling Technology	Cat#9717
Acetyl-Histone H3(Lys27) Rabbit mAb	Cell Signaling Technology	Cat#8173
Tri-Methyl-Histone H3(Lys27) Rabbit mAb	Cell Signaling Technology	Cat#9733
Ubiquityl-Histone H2A(Lys119) Rabbit mAb	Cell Signaling Technology	Cat#8240
DYKDDDDK Tag Rabbit mAb	Cell Signaling Technology	Cat#14793
Anti-DDDDK-tag mAb	MBL	Cat#M185
Anti-Myc-tag mAb	MBL	Cat#M192
FLAG® M2 Mouse mAb	Sigma-Aldrich	Cat#F1804
Myc-tag mouse mAb	Abmart	Cat#M20002
HA-tag mouse mAb	Abmart	Cat#M20003
GFP-tag mouse mAb	Abmart	Cat#M20004
Beta Actin Antibody	Sharebio	Cat#AB0035
α-tubulin Mouse Monoclonal Antibody	signalway antibody	Cat#38059
HRP-conjugated Goat anti-Rabbit IgG	CW-Biotech	Cat#CW0103S
HRP-conjugated Goat anti-Mouse IgG	CW-Biotech	Cat#CW0102S
GFP mouse Ab	Beyotime	Cat#AG281
HRP-conjugated Goat anti-Rabbit IgG	Beyotime	Cat#A0208
Rabbit monoclonal anti-DDDDK tag antibody	Abcam	ab205606
Rabbit monoclonal anti-GAPDH, HRP-linked antibody	Cell Signaling	5174
Anti-rabbit IgG, HRP-linked antibody	Cell Signaling	7074
Critical Commercial Assays		
T3 Super PCR Mix	Tsingke	Cat#TSE030
Trelief™ SoSoo Cloning Kit Ver.2	Tsingke	Cat#TSV-S2
MightyAmp™ DNA Polymerase Ver.3	TAKARA	Cat#R076B
PrimeScript™ 1st Strand cDNA Synthesis Kit	TAKARA	Cat#6110B
CV16-ZeroBackgroundpTOPO-Blunt Cloning Kit	Aidlab	Cat#CV0404
NEBuilder® HiFi DNA Assembly Master Mix	NEB	Cat#E2621S
mMESSAGE mMACHINE SP6 Transcription Kit	Invitrogen	Cat#AM1348
MEGAshortscript™ T7 Kit	Invitrogen	Cat#AM1354
MAXIscript SP6/T7 Transcription Kit	Invitrogen	Cat#AM1322
MEGAclear Transcription Cleanup Kit	Invitrogen	Cat#AM1908
Poly(A) Tailing Kit	Invitrogen	Cat#AM1350
SigmaSpin Sequencing Reaction Clean-up	Sigma-Aldrich	Cat#S5059
SMARTer® Stranded Total RNA-Seq Kit v2	TaKaRa Bio	634417
NEBNext® Multiplex Small RNA Library Prep Kit for Illumina®	NEB	E7560S
Chemiluminescent Nucleic Acid Detection Module Kit	ThermoFisher Scientific	89880
RiboMinus™ Eukaryote System v2	Invitrogen	A15026
RNA Clean & Concentrator-5	Zymo Research	R1014
Experimental models: Cell Lines		
Human: HEK293T	TaoZhong Lab in Fudan university	N/A
Experimental models: Organisms/Strains		
*mbd5* ^Δ29^, *mbd5*^Δ42^, *mbd5*^ins25^, *mbd5*^ΔMBD^ mutants	This study	N/A
*mbd6* ^Δ13^, *mbd6*^Δ22^ mutants	This study	N/A
*mbd5* ^Δ29^ & *mbd6^+/^*^Δ22^ mutants	This study	N/A
Tg(hsp70l:FLAG*mbd5*^iso2^-E2A-EGFP)	This study	N/A
Recombinant DNA		
Plasmid: pT2-hsp70l:FLAG*mbd5*^iso2^-E2A-EGFP	This study	N/A
Plasmid: pCMV6-XL4 ASXL1 (p.Y591X) 3x Flag	Kweon et al., 2019	N/A
Plasmid: pcDNA3.0–3x Flag HmMBD5^iso2^	This study	N/A
Plasmid: pcDNA3.0-ZfAsxl1-myc	This study	N/A
Plasmid: pcDNA3.0–3x Flag ZfMbd5^iso2^	This study	N/A
Plasmid: pcDNA3.0-EGFP-ZfMbd5^iso2^-MBD	This study	N/A
Plasmid: pcDNA3.0-EGFP-ZfMbd5-PWWP	This study	N/A
Plasmid: pcDNA3.0-ZfBap1-HA	This study	N/A
Oligonucleotides(5′-3′)		
Control MO: CCAGCTATTTTACACCATTAATATT	GeneTools	N/A
*mbd5 5′*UTR MO:TATGGTCCAGTGCTAAACTCCTGCA	GeneTools	N/A
p53 MO: GCGCCATTGCTTTGCAAGAATTG	GeneTools	N/A
Control RNA oligo for ELISA experiments: 5′-AlexaFluor594- UAUGACCUGCGG UAUGACCUGCGGUAUGACCUGCGG-3′	This paper	N/A
Methylated RNA oligo for ELISA experiments: 5′-AlexaFluor594- UAUGAC(m^5^C)UGCGG UAUGAC(m^5^C)UGCGGUAUGAC(m^5^C)UGCGG-3′	This paper	N/A
Software		
ImageJ	Schindelin *et al.*(2015)	https://imagej.nih.gov/ij/
CHOPCHOP	Labun *et al.*(2019)	http://chopchop.cbu.uib.no/
Cas-OFFinder	Bae S et al.(2014)	http://www.rgenome.net/cas-offinder/
GraphPad Prism 9	GraphPad Software	https://www.graphpad.com/scientific-software/prism/
R v3.3.0 /Bioconductor v3.3	R core team	https://www.bioconductor.org/
STAR	Dobin *et al.* (2012)	https://github.com/alexdobin/STAR
Trimmomatic	Bolger *et al.* (2014)	https://github.com/usadellab/Trimmomatic
SAMtools	Li *et al.* (2009)	http://www.htslib.org/
Cutadapt	Martin *et al.* (2011)	https://github.com/marcelm/cutadapt
HISAT2	Kim *et al.* (2015)	N/A

## Experimental model and subject details

### Animal subjects

Experiments were carried out in accordance with protocols approved by the Institutional Animal Care and Use Committee. Zebrafish embryos were treated with 0.003% 1-phenyl-2-thiourea (PTU) in E3 medium to prevent pigment formation. 3–9 months-aged wild type or *mbd5* mutant lines (generated in this study) were used for breeding or adult behavior analyses. Developmental stages were determined according to their morphology (16).

## Method details

### Generation of zebrafish mutants by the CRISPR-Cas9 system

The detailed procedures of gene editing with CRISPR/Cas9 in zebrafish were described previously (17,18). We designed sgRNAs via an online website (https://zlab.bio/guide-design-resources). Two sgRNA target sites were selected for *mbd5*, which targets Exon 2 and Exon 4 respectively and one sgRNA target site was selected for *mbd6* KO. Cas9 mRNA and sgRNAs were synthesized with the mMESSAGE mMACHINE T3 Transcription Kit (AM1348, Invitrogen, United States) and MEGAshortscript™ T7 Kit (AM1354, Invitrogen, United States), respectively. Mixtures composed of 300 pg Cas9 mRNA and 25 pg sgRNA were microinjected into one-cell stage fertilized embryos, which were raised and screened for germline transmission. After outcrossing to wildtype AB background for several generations, several alleles of zebrafish *mbd5* and *mbd6* mutants were established and maintained. The sequences of the target sites and primers used to genotype were listed in Supplemental Table S1.

### Generation of *tg(zhsp70l:3 × Flag-mbd5-E2A-EGFP)* zebrafish and heat shock induction of FLAG-tagged Mbd5

The zhsp70l-E2A cassette was synthesized (GENEWIZ, Suzhou, China) and cloned upstream of EGFP in plasmid pT2KXIGΔin to replace xenopus ef1α and β-globin intron. Then 3 × Flag fused *mbd5^iso2^* or *mbd5^iso1^* were subcloned between zhsp70l and E2A elements. Finally, Tol2 transposase mRNA (19) and the plasmid construct were co-injected into one-cell-stage fertilized eggs, followed by raising, screening and propagation as previously described (20).

To induce FLAG-tagged Mbd5 for biochemical and immunocytochemical experiments, heat shock was carried out at 32 hpf, 48 hpf and 72 hpf, by placing embryos and larvae in 120 ml E3 medium in a glass beaker, in a water bath at 38°C, for 1 h.

### Whole mount *in situ* hybridization (WISH) and immunostaining

Zebrafish embryos of different stages were fixed in 4% paraformaldehyde overnight at 4°C. For WISH, the fixed embryos were subjected to gradient dehydration and rehydration and the rest of the procedures were performed as previously described (21). To generate RNA probes, PCR products of *mbd5*, *vglut2a*, *gad1b*, *th1*, *glyt2*, *nestin*, *neurod1*, *cntn2* were amplified from cDNAs and subcloned into pTOPO-blunt vector (CV1601, Aidlab), and the primers were listed in Supplemental Table S2. After sequence validation, probe plasmids were linearized with restriction endonucleases, and Digoxigenin-labeled RNA probes were transcribed with the linearized DNA template using MAXIscript SP6/T7 Transcription Kit (AM1322, Invitrogen). The signal was detected with BM-purple (11442074001, Roche), and staining process was visually examined until the signal reached an optimal level. The WISH images were taken with Leica M205FA microscope. For Immunostaining, after gradient dehydration and rehydration, fixed embryos required extra antigen retrieval (Incubation in PH 9.5 Tris–HCl for 15 min at 70°C). Primary antibodies were incubated at 4°C overnight and secondary antibodies were incubated at room temperature for 4 hrs. Antibodies used in this study included the Anti-DDDDK-tag (FLAG tag) mAb (1:500, M185, MBL) and donkey anti-mouse Alexa Fluor 647 (1:1000, A31571, Invitrogen).

### Zebrafish behavior

The DanioVision apparatus (Noldus, Netherlands) combined with a temperature controller, a light-tight chamber, an infrared camera and an automated VideoTrack software was used to track larval and adult zebrafish behavior. The traveled distance and velocity of larval and adult zebrafish were measured to compute general locomotor activity. For larvae, 24 larval zebrafish at ∼6 dpf were transferred to 24-well plates individually and placed in the monitor chamber for behavioral recording. In the total 15 min of recording, the first 5 min was an acclimation phase, and the remaining time was used for data analysis. Activity was quantified using EthoVision XT software. For adults, male zebrafish were allowed to freely swim inside a 10 cm × 10 cm × 15 cm tank, one animal at a time in a novel-tank assay. In addition to measuring locomotor activity, we divided the tank into three equal virtual zones: top, middle, and bottom. Time spent in different zones was used to evaluate anxiety-associated behaviors, as previously described (22).

### Drug treatment

PTZ (P6500, Sigma-Aldrich) was dissolved in E3 medium at 100 mM and stored at –20°C. Zebrafish at 6 dpf were treated with 2.5 mM PTZ and placed in the DanioVision apparatus for behavior recording. FAC (F5879, Sigma-Aldrich) was dissolved in E3 medium at 50 mM and stored at 4°C. zebrafish were treated with 1 mM fresh FAC from 3 to 6 dpf every day prior to behavior analysis. DFO (D9533, Sigma-Aldrich) was dissolved in E3 medium at 150 mM and stored at –20°C. Zebrafish were treated with 0.5 mM DFO from 3 to 6 dpf, replenished every day, prior to behavior analysis.

### Gene expression analysis

For quantitative Real-time PCR (qRT-PCR) analysis, total RNAs were extracted using the TRIzol reagent (T9424, Sigma-Aldrich), and cDNA was synthesized with PrimeScript™ 1st Strand cDNA Synthesis Kit (6110B, TaKaRa) according to the manufacturer's instructions. qPCR incubation was done in LightCycler® 480 Instrument II (Roche) with FastStart™ Universal SYBR^®^ Green Mix (ROX) (4913914001, Roche). Finally, the delta CT method was used to calculate the expression levels. Primers used were described in Supplemental Table S3.

For RNA-seq of KD and KO at 52 hpf as well as RNA-seq of KO and Tg at 7 dpf, ∼30 larvae were collected in a group. After 3× washes with PBS, TRIzol reagents were added and frozen at –80°C. RNA extraction, quality inspection, library construction, and analysis of differentially expressed genes were performed by GENEWIZ, and Next-Generation Sequencing was performed on the Illumina 10X Genomics platform. To identify the commonly regulated genes between different groups, we did GO and KEGG analysis on the OmicShare platform (https://www.omicshare.com/) according to the set threshold.

For RNA-seq of KD at 76 hpf, total RNA was isolated using the TRIzol reagent, and ribo-minus RNA libraries were prepared using SMARTer® Stranded Total RNA-Seq Kit v2-Pico Input Mammalian (TaKaRa) according to the manufacturer's protocol. Three biological replicates were sequenced on an Illumina NovaSeq 6000 sequencer (100 bp, single end) for each treatment. Raw reads were trimmed with Trimmomatic-0.39 (23), then aligned to zebrafish genome and transcriptome (danRer11) using HISAT (version 2.2.1) (24) with ‘–rna-strandness R’ parameters. Annotation files (RefSeq, 2020-04-01, in gtf format) were downloaded from NCBI. Reads on each NCBI annotated gene were counted using HTSeq (version 1.12.4) (25) and then differentially expressed genes were called using DESeq2 package (version 1.26.0) in R (26). Differentially expressed genes were identified as genes with at least 10 read counts in at least two samples with *P* adjusted <0.05.

### Biotin labelling of nucleic acids co-immunoprecipitated with zebrafish Mbd5

The biotin labelling of nucleic acids was performed as previously described (27). 76 hpf *Tg[zhsp70l:FLAGmbd5^iso2^-E2AGFP]* larval zebrafish were UV crosslinked and flash-frozen in liquid nitrogen. Pellets were resuspended in cold eCLIP lysis buffer (50 mM Tris–HCl pH 7.4, 100 mM NaCl, 1% NP-40 (Igepal CA630), 0.1% SDS, 0.5% sodium deoxycholate (protect from light), 1 × Halt™ Protease and Phosphatase Inhibitor Cocktail, in RNase/DNase free H_2_O). Pellets were lysed by rotating at 4°C for 15 min after passing through a 26 G needle (BD Biosciences). Larval extracts were sonicated on a bioruptor (Diagenode) with 30 s on/30 s off for 5 cycles. 4 U/μl RNase I (Ambion) or 4 U/μl DNase I (Invitrogen) treatment was performed on suspensions at 37°C for 5 min. Lysates were cleared by centrifugation at 21 000 g for 15 min at 4°C on a benchtop centrifuge. Supernatants were applied to Flag-antibody (Abcam) conjugated protein A beads (Invitrogen) and left overnight at 4°C on an end-to-end rotor. Beads were washed twice with ice-cold High Salt Wash Buffer (50 mM Tris–HCl pH 7.4, 1 M NaCl, 1 mM EDTA, 1% NP-40, 0.1% SDS, 0.5% sodium deoxycholate, in RNase/DNase free H_2_O) and three times with ice-cold Wash Buffer (20 mM Tris–HCl pH 7.4, 10 mM MgCl_2_, 0.2% Tween-20, in RNase/DNase free H_2_O). Protein–nucleic acid complex conjugated to the beads were dephosphorylated by FastAP (Thermo Scientific) and T4 PNK (Thermo Scientific). Beads were washed twice with ice-cold High Salt Wash Buffer and three times with ice-cold Wash Buffer. Protein-nucleic acid complex conjugated to the beads were biotinylated with Biotinylated Cytidine (Bjs)phosphate (Jena Bioscience). Beads were washed twice with ice-cold High Salt Wash Buffer and three times with ice-cold Wash Buffer. Washed beads were subjected to SDS-PAGE electrophoresis and membrane transfer. The biotin signal was blotted with Chemiluminescent Nucleic Acid Detection Module kit (Thermo Scientific).

### Electrophoretic mobility shift assay

Recombinant His-tagged Mbd5-GFP is purified with BL21(DE3) competent *Escherichia coli* expression. Different concentrations of Mbd5-GFP were mixed with 100 ng denatured RNA or DNA probe in 1 × binding buffer (20 mM HEPES pH 7.5, 40 mM KCl, 10 mM MgCl_2_, 0.1% Triton X-100, 10% glycerol and 1 × RNaseOUT Recombinant Ribonuclease Inhibitor). The probe–protein mixture was incubated on ice for 30 min. The mixtures were loaded to a 4–20% Novex™ TBE Gel (Invitrogen). After gel running at 4°C in 0.5 × TBE, the gel was stained with SYBR Gold (Invitrogen) at room temperature for 30 min before imaging with a GelDoc imaging system (Bio-Rad). Individual *K*_D_ values were determined from a regression equation *Y* = Max × [P]/(*K*_D_ + [P]). *Y* is the fraction of probe bound at each protein concentration. Fraction bound is determined from the background-subtracted signal intensities using the expression: bound/(bound + unbound). [P] is protein concentration in each sample. Max is the band intensity of unbound probe with protein concentration 0.

### Cross-linking immunoprecipitation sequencing (CLIP-seq) and analysis

76 hpf larval zebrafish were UV crosslinked and flash-frozen in liquid nitrogen. Pellets were thawed on ice and resuspended in 3 volume of ice-cold CLIP lysis buffer (50 mM HEPES pH 7.5, 150 mM KCl, 2 mM EDTA, 0.5% (v/v) NP-40, 0.5 mM DTT, 1 × Halt™ Protease and Phosphatase Inhibitor Cocktail, 1 × RNaseOUT Recombinant Ribonuclease Inhibitor). Pellets were lysed by rotating at 4°C for 15 min after passing through a 26 G needle (BD Biosciences). Embryo suspensions were sonicated on a bioruptor (Diagenode) with 30 s on/30 s off for 5 cycles. Lysates were cleared by centrifugation at 21 000 g for 15 min at 4°C on a benchtop centrifuge. Supernatants were applied to Flag-antibody (Abcam) conjugated protein A beads (Invitrogen) and left overnight at 4°C on an end-to-end rotor. Beads were washed extensively with 1 ml wash buffer (50 mM HEPES pH 7.5, 300 mM KCl, 0.05% (v/v) NP-40, 1 × Halt™ Protease and Phosphatase Inhibitor Cocktail, 1 × RNaseOUT Recombinant Ribonuclease Inhibitor) at 4°C for 5 times. Protein-RNA complex conjugated to the beads were treated by 8 U/μl RNase T1 (Thermo Scientific) at 22°C for 10 min with shaking. Input samples are digested in parallel. Then input and IP samples were separated on an SDS-PAGE gel and gel slices at corresponding size ranges were treated by proteinase K (Invitrogen) elution. RNA was recovered with TRIZol reagent (Invitrogen). Then T4 PNK (Thermo Scientific) end repair was performed with purified RNA before library construction with NEB small RNA kit (NEB). Libraries were pooled and sequenced on a NovaSeq 6000 sequencer with paired-end mode 50 bp setting. For the CLIP fragment-qPCR to assay Mbd5 binding on specific transcripts, qPCR analysis was carried out following CLIP using the primers as listed in Supplemental Table S4.

For western blot validation of protein immunoprecipitation, protein samples were prepared from respective zebrafish embryos by lysis in RIPA buffer (Invitrogen) containing 1 × Halt™ Protease and Phosphatase Inhibitor Cocktail (ThermoFisher Scientific). Protein concentration was measured by NanoDrop 8000 Spectrophotometer (ThermoFisher Scientific). Lysates were boiled at 95°C with 1 × loading buffer (Biorad) for five min. Denatured protein was loaded into 4–12% NuPAGE Bis–Tris gel and transferred to PVDF membranes (Life Technologies). Membranes were blocked in PBST with 3% BSA (MilliporeSigma) for 30 min at room temperature, incubated in a diluted primary antibody solution at 4°C overnight, washed and incubated in a dilution of secondary antibody conjugated to HRP for 1 h at room temperature. Protein bands were detected using SuperSignal West Dura Extended Duration Substrate kit (ThermoFisher Scientific) on FluroChem R (Proteinsimple).

Low quality reads were removed to reduce false positive rate due to sequencing error using fastq_quality_filter with ‘-q 20’ parameter. Adapter and reads shorter than 15 nt were removed using fastx_clipper with ‘-a -l 15’ parameter. To remove PCR duplicates, reads were collapsed using fastx_collapser (FASTX Toolkit 0.0.14). Cleaned reads were aligned to zebrafish genome (danRer11) using bowtie (version 1.0.0) (28) with ‘-v 3 -m 10 –best –strata’ parameters. Mapped reads were separated by strands with samtools (version 1.9) (29) and peaks on each strand were called using MACS (version 2) (30) with parameter ‘-nomodel, –keep-dup all, -g 1.4e9, –tsize 60 -extsize 30′ separately. Significant peaks with *q* < 0.01 identified by MACS2 were considered. Peaks identified in at least two biological replicates using bedtools (v.2.26.0) (29) and were used in the following analysis.

### Quantitative analysis of RNA modification levels of CLIP RNA

76 hpf zebrafish larvae were UV crosslinked and flash-frozen in liquid nitrogen. Pellets were thawed on ice and resuspended in 3 volume of ice-cold CLIP lysis buffer (50 mM HEPES pH 7.5, 150 mM KCl, 2 mM EDTA, 0.5% (v/v) NP-40, 0.5 mM DTT, 1 × Halt™ Protease and Phosphatase Inhibitor Cocktail, 1 × RNaseOUT Recombinant Ribonuclease Inhibitor). Pellets were lysed by rotating at 4°C for 15 min after passing through a 26 G needle (BD Biosciences). Embryo suspensions were sonicated on a bioruptor (Diagenode) with 30 s on/30 s off for 5 cycles. Lysates were cleared by centrifugation at 21 000 g for 15 min at 4°C on a benchtop centrifuge. Supernatants were applied to Flag-antibody (Abcam) conjugated protein A beads (Invitrogen) and left overnight at 4°C on an end-to-end rotor. Beads were washed extensively with 1 ml wash buffer (50 mM HEPES pH 7.5, 300 mM KCl, 0.05% (v/v) NP-40, 1 × Halt™ Protease and Phosphatase Inhibitor Cocktail, 1 × RNaseOUT Recombinant Ribonuclease Inhibitor) at 4°C for 5 times. Then input and IP samples were treated by proteinase K (Invitrogen) to release crosslinked RNA. RNA was recovered with TRIZol reagent (Invitrogen). Then ribosomal RNA was removed by using RiboMinus™ Eukaryote System v2 (Invitrogen) with size-selection of RNA Clean & Concentrator-5 (Zymo Research). Ribo-minus input and IP RNA were digested to single nucleosides by nuclease P1 (MilliporeSigma) and FastAP (Thermo Scientific). The samples were then filtered (0.22 mm, Millipore) and injected into a C18 reverse phase column coupled online to Agilent 6460 LC–MS/MS spectrometer in positive electrospray ionization mode. The nucleosides were quantified by using retention time and the nucleoside to base ion mass transitions (268-to-136 for A; 256-to-150 for m^5^C). Quantification was performed by comparing with the standard curve obtained from pure nucleoside standards running with the same batch of samples.

### *In vivo* co-immunoprecipitation (co-IP) and LC–MS/MS

After heat-shock, *Tg(zhsp70l:mbd5^iso2^-E2A-EGFP)* zebrafish larvae were collected, washed with PBS, and deyolked in the deyolk buffer (55 mM NaCl, 1.8 mM KCl, 1.25 mM NaHCO_3_) and homogenized in the RIPA lysis buffer (P0013B, Beyotime) containing protease inhibitor cocktail (K1007, APExBio). After incubation on ice for 30 min, sonication was carried out for another 2–5 min until most of the tissues were dissolved. The protein lysate was obtained by centrifugation at 14 000 g for 20 min at 4°C. The supernatants were incubated with anti-Flag M2 Affinity Gel (A2220, Millipore) or anti-IgG Agarose (A0919, Sigma-Aldrich) as a control at 4°C overnight. The beads were washed with the RIPA lysis buffer five times, and then boiled in SDS loading buffer (P0015L, Beyotime) for immunoblot. IP samples’ reduction, alkylation, digestion, and subsequent LC–MS/MS were performed by LuMing Biotech (Shanghai). For total protein western blot, supernatants were directly mixed with the SDS loading buffer and boiled at 95°C for 10 min. For histone western blot, Nuclear and Cytoplasmic Protein Extraction kit (P0027, Beyotime) was used to extract nuclear proteins. Antibodies applied in western-blot analysis include anti-DDDDK-tag mAb (1:5000, M185, MBL), Rabbit anti Beta Actin Antibody (1:3000, AB0035, Share-bio), α-tubulin Mouse Monoclonal Antibody (1:5000, 38059, SAB), Histone H3 Rabbit mAb (1:1000, 9717, CST), Tri-Methyl-Histone H3(Lys27) Rabbit mAb (1:2000, 9733, CST), Acetyl-Histone H3(Lys27) Rabbit mAb (1:2000, 8173, CST) and Ubiquityl-Histone H2A(Lys119) (1:2000, 8240, CST).

### Cell culture, co-immunoprecipitation and western blotting

For co-immunoprecipitation, we constructed a series of expression plasmids based on pcDNA3.0 Vector which included Flag-fused ZfMbd5^iso2^ FL, GFP-tagged ZfMbd5^iso2^-MBD, GFP-tagged ZfMbd5^iso2^-PWWP, HA-fused ZfBap1 and myc-fused ZfAsxl1 (1–600 aa). The human MBD5 plasmid was a gift from Drs Mengqi Zhou and Jinrong Meng (6). The human AXL1 (Y591X) plasmid was a gift from Dr Douglas Feldman (31). HEK293T cells were co-transfected with these plasmids using Lipofectamine2000 Transfection Reagent (11668019, Invitrogen). After 2–3 days of incubation, the transfected cells were lysed with NETT buffer (50 mM Tris–HCl, 100 mM EDTA, 150 mM NaCl, 1% TritonX-100) containing protease inhibitor cocktail and incubated on ice for 10 min. The cell lysates were sonicated for 2–4 min until the protein lysates were clear. The supernatants were obtained by centrifugation at 14 000 g for 20 min at 4°C and incubated with anti-tag antibodies at 4°C overnight. The equilibrated Protein A/G PLUS-Agarose (Sc-2003, Santa Cruz) was added into protein lysate mixture and incubated for another 4 h in the next day. The agarose precipitates were washed with NETT buffer five times and boiled in SDS loading buffer at 95°C for 10 min. After centrifugation for another 5 min, the supernatants were used for western blot analysis. Antibodies used in this part include anti-DDDDK-tag mAb (IP, 1:200, M185, MBL), GFP-tag mouse mAb (IP, 1:250, WB, 1:3000, M20004, Abmart), Myc-tag mouse mAb (WB, 1:3000, M20002, Abmart) and HA-tag mouse mAb (WB, 1:3000, M20003, Abmart).

### Statistical analysis

Gray-value of protein blots in western blots were manually calculated using ImageJ software. GraphPad Prism7 was used for data analysis. For single factor, two groups were analyzed by Student's *t*-test, one-way ANOVA with Dunnett's multiple comparisons test were performed for three or more groups of samples. For two experimental factors, two-way ANOVA with Tukey's multiple comparisons were analyzed for two or more groups of samples. Other analysis methods were indicated in each section.

## Results

### Morpholino antisense oligonucleotide-mediated knockdown of *mbd5* disrupts embryonic development in zebrafish

**Figure 1.**
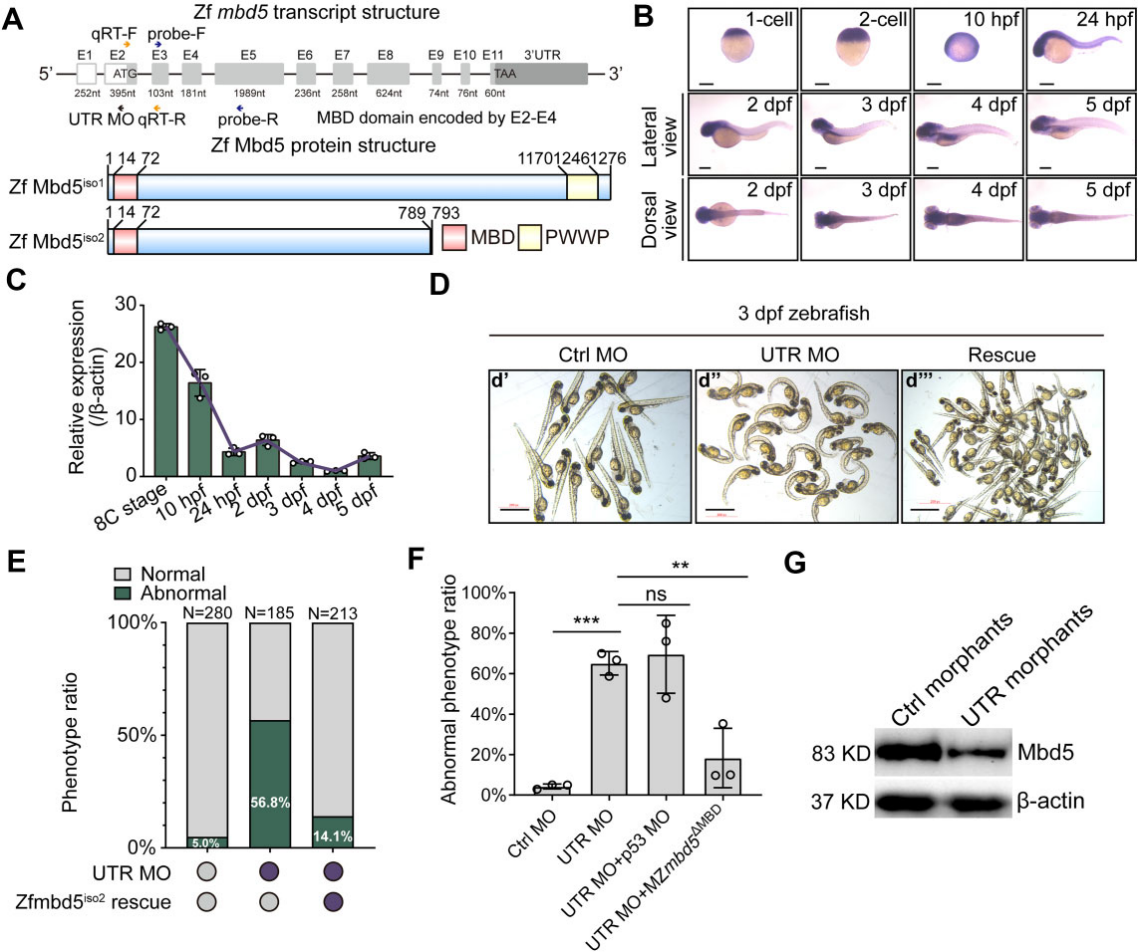
*mbd5* is essential for zebrafish embryonic development. (**A**) Schematic of the zebrafish *mbd5* gene structure and two isoforms. (**B**) Whole mount *in situ* hybridization of *mbd5* from 1-cell stage to 5 days post fertilization (dpf). (**C**) Quantitative Real-Time PCR (qRT-PCR) analysis of *mbd5* expression from 8-cell stage to 5 dpf. The expression of *mbd5* at 4 dpf was normalized as 1 to calculate relative levels of other groups. β-actin was used as the internal standard. (**D**) Images of 3 dpf larval zebrafish injected with ctrl MO (**d’**), MO targeting the 5′untranslated region of *mbd5* (5′ UTR MO) (**d’’**), which showed ventrally curved body and pericardial edema phenotypes that were partially rescued by co-injection of zebrafish *mbd5* isoform2 (*mbd5*^iso2^) mRNA (**d’’’**). (**E**) Phenotypic ratios induced by control MO, 5′ UTR MO and 5′ UTR MO con-injected with the *mbd5*^iso2^ mRNA. (**F**) Quantification of abnormal phenotypic ratios in different experimental groups shows that 5′ UTR MO-induced phenotypes are not due to cell death triggered by p53-mediated apoptosis and are not detected in *mbd5*^ΔMBD^ CRISPR knockout mutants injected with the 5′ UTR MO. Data are collected from three independent experiments and analyzed using two-way ANOVA followed by Dunnett's multiple comparison test. Mean ± S.D. is shown. *N* = 71 (Ctrl MO), *N* = 56 (UTR MO), *N* = 62 (UTR MO + p53 MO), *N* = 57 (UTR MO + MZmbd5 ^ΔMBD^). Ns, not significant; ** *P* < 0.01; *** *P* < 0.001. (**G**) Western blot using a custom Mbd5 antibody shows reduced Mbd5 protein levels in the 5′ UTR morphants compared to controls. Scale bar, 200 μm.

To understand the role of *mbd5* in zebrafish, we first defined its spatiotemporal expression pattern using whole mount *in situ* hybridization (WISH). In both zebrafish and humans, two isoforms of Mbd5 exist. The longer isoform 1 contains the N-terminal MBD domain and the C-terminal proline–tryptophan–tryptophan–proline (PWWP) domain. The PWWP domain is found in eukaryotic chromatin-associated proteins (32) including DNA methyltransferases (33). The shorter isoform 2 only has the MBD domain (Figure 1A). Using an anti-sense probe that hybridizes to both isoforms, we detected strong maternal expression at 1–2 cell stage and ubiquitous expression at 10 h post fertilization (hpf) and 24 hpf, whereas the sense control probe gave no signal. As development progressed, *mbd5* expression became enriched in the brain (Figure 1B). Real-Time PCR (qRT-PCR) analysis quantified the relative expression of *mbd5* transcripts at multiple developmental stages (Figure 1C).

Using translation-blocking morpholino antisense-oligonucleotides (MO), we knocked down *mbd5*. Ventrally curved body and pericardial edema were observed in the *mbd5* morphants. To determine whether this developmental phenotype was specific to Mbd5 knockdown, we performed mRNA rescue experiments. The *mbd5* isoform 1 mRNA, when over-expressed in wildtype (WT), had a gain-of-function phenotype resulting in embryonic deformity, and was therefore not used in this study. The zebrafish *mbd5* isoform2 (*mbd5*^iso2^) mRNA was able to rescue the developmental deficits of *mbd5* morphants (Figure 1D, E). Since p53 activation is sometimes a nonspecific off-target effect in MO knockdown technologies (34), we co-injected *mbd5* 5′ UTR MO and p53 MO into 1 cell-stage embryos and found no obvious phenotypic ratio reduction compared to the 5′ UTR MO alone (Figure 1F), suggesting no involvement of p53 activation in the *mbd5* morphants. Furthermore, the 5′ UTR MO did not induce any phenotypes in *mbd5*^ΔMBD^ CRISPR knockout (KO) mutants (See later sections for the description of KO) (Figure 1F), again demonstrating the specificity of the MO in interfering with *mbd5* gene activity. The MO knockdown efficacy was further validated by western blotting (Figure 1G). Taken together, these results indicate that the developmental deficits in *mbd5* morphants can be attributed to specific knockdown of Mbd5. Thus, MBD5 plays an essential role in vertebrate embryonic development.

### Zebrafish *mbd5* germline mutants generated by CRISPR/Cas9 editing are adult viable and exhibit behavioral abnormalities

**Figure 2.**
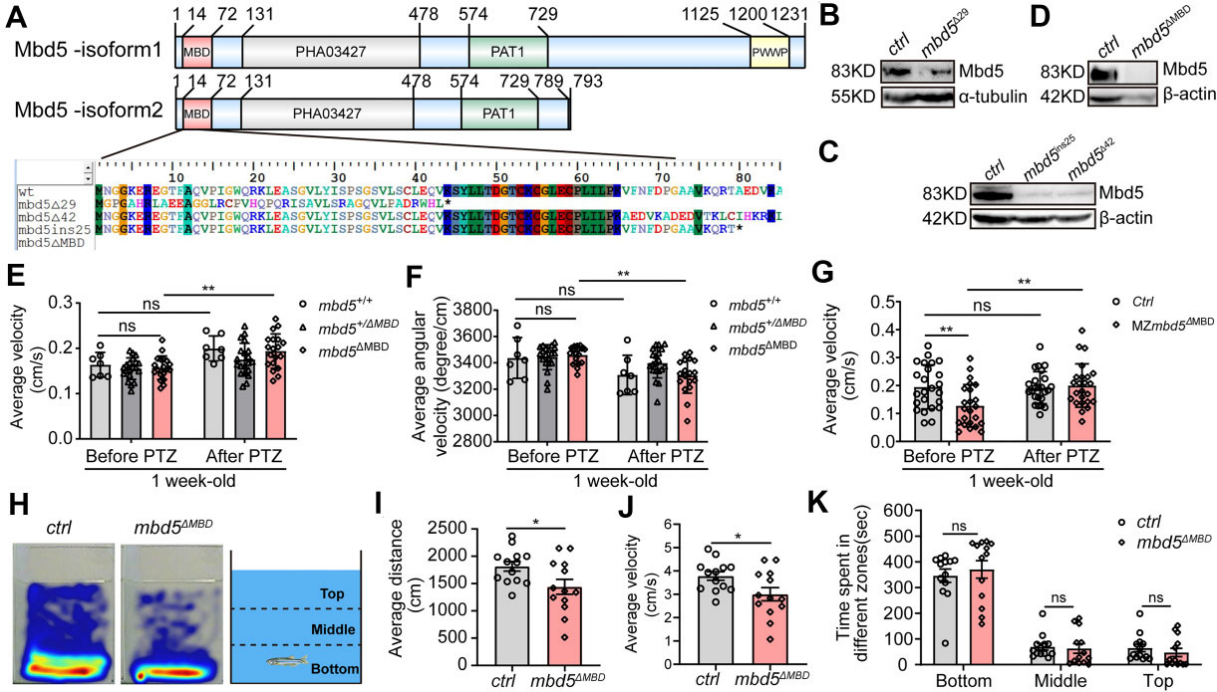
Germ-line knockouts of *mbd5* are adult viable and suffer from behavioral abnormalities. (**A**) Predicted amino acid sequences of wild type and *mbd5* mutant alleles. (**B**–**D**) Western-blot shows reduced Mbd5 protein in different *mbd5* mutant alleles. (**E**, **F**) Locomotor behavioral analysis of the 7 dpf progeny from *mbd5^+/^*^ΔMBD^ in-crosses upon treatment with 2.5mM Pentylenetetrazol (PTZ). The following numbers of individuals were obtained from the in-cross: *mbd5^+/^*^+^, *N* = 10; *mbd5^+/^*^ΔMBD^, *N* = 20; *mbd5*^ΔMBD^, *N* = 18. because some larvae did not move after PTZ treatment, the following numbers were used for data analysis: *mbd5^+/^*^+^, *N* = 7; *mbd5^+/^*^ΔMBD^, *N* = 20; *mbd5*^ΔMBD^, *N* = 17. (**G**) Locomotor defects in maternal-zygotic *mbd5*^ΔMBD^ mutants and hyperactivity measured by average velocity after 2.5 mM PTZ treatment, *N* = 24. (**H**–**K**) Representative heat maps of adult ctrl (H, left) and *mbd5*^ΔMBD^ (H, middle) zebrafish in a novel-tank assay virtually divided to top, middle and bottom zones (H, right). Locomotor behavioral analysis shows that the average distance moved (I) and average velocity (J) are significantly reduced in *mbd5*^ΔMBD^ mutants comparing to controls. The time spent in different zones (K) shows no difference between *mbd5*^ΔMBD^ mutants and controls. *N* = 13. Each value represents mean ± SD, ns, no significance, *P< 0.05, ** *P*< 0.01, *** *P*< 0.001.

To further decipher the biological function of *mbd5*, we generated homozygous *mbd5* mutants using the CRISPR/Cas9 genome editing technology (Figure 2 and Supplemental Figure S1). Two sgRNAs targeting Exon 2 and Exon 4 respectively were designed and transcribed *in vitro*, followed by co-injection with the Cas9 mRNA into one-cell embryos. We obtained four mutant alleles, *mbd5*^Δ29^, *mbd5*^ins25^, *mbd5*^Δ42^ and *mbd5*^ΔMBD^, with 29 base pairs (bp) deletion, 25 bp insertion, 42 bp deletion and 1649 bp deletion of the entire MBD domain coding sequence, respectively (Supplemental Figure S1A–D). Premature stop codons were predicted in *mbd5*^Δ29^ and *mbd5*^ins25^ mutants, yielding truncated proteins of 43 and 79 amino acids (aa) respectively. *mbd5*^Δ42^ and *mbd5*^ΔMBD^ alleles were predicted to yield 14 aa in-frame deletion and whole MBD domain deletion including the translation start site, respectively (Figure 2A). Western-blot analysis further verified the decrease of Mbd5 protein in all four alleles; Some residual Mbd5 proteins are possibly due to maternal products (Figure 2B–D). *mbd5*^ΔMBD^ or *mbd5*^Δ29^ mutant lines were used in subsequent studies.

The *mbd5* mutants displayed no morphological defects during development. Since disrupted balance between excitatory and inhibitory neurotransmitter signaling is observed in ASD and epilepsy (35), we examined whether such balance might be disrupted in the *mbd5* mutants by analyzing the expression of excitatory neurotransmitter marker vesicular glutamate transporter (*vglut2a*) (Supplemental Figure S1E^A-C’^), the inhibitory neurotransmitter marker glutamate decarboxylase 1b (*gad1b*) (Supplemental Figure S1E^D-F’^), as well as tyrosine hydroxylase (*th*) (Supplemental Figure S1E^G-H’^) and glycine transporter (glyt2) (Supplemental Figure S1E^I-K’^). These neuronal markers all appeared normal in the *mbd5*^ΔMBD^ mutants. Furthermore, we examined the expression of neural progenitor cell markers and neuronal differentiation markers, including *neurod1* (neuronal differentiation 1), *nestin* (a member of intermediate filament protein family), and *cntn2* (transiently expressed axonal glycoprotein) and found no significant difference between mutants and their wildtype siblings (Supplemental Figure S1E^L-Q’^). No obvious changes were found for *mbd5* transcripts either (Supplemental Figure S1E^R-S’^).

Given the grossly normal morphology, we next examined whether functional deficits might exist in the *mbd5* mutants. It has been observed that GABAergic signaling deficits increase the susceptibility to pentylenetetrazol (PTZ)-induced seizures (36). We therefore treated the in-cross progeny of *mbd5^+/^*^ΔMBD^ with 2.5 mM PTZ followed by behavioral analysis. At 7 days post fertilization (dpf), while zygotic *mbd5*^ΔMBD^ mutants showed grossly normal motor behavior, they displayed increased PTZ sensitivity, as evidenced by increased velocity and reduced angular velocity (suggesting difficulty in turn maneuver) in comparison to WT siblings (Figure 2E, F). Maternal-zygotic *mbd5*^ΔMBD^ mutants displayed both reduced baseline locomotor activity and increased sensitivity to low-dose PTZ (Figure 2G). Adult *mbd5*^ΔMBD^ mutants also exhibited reduced locomotor activity (Figure 2H-J), while anxiety-like behaviors were no different when compared to age-matched controls in a novel-tank assay (Figure 2K). These observations uncover normal morphology but functional deficits at the behavioral level in the *mbd5* germline mutants. The phenotypic differences between *mbd5* KD versus KO are likely due to genetic compensation in the germline KOs, which have been previously observed for other genes (37).

### Mbd6 partially compensates for the loss of Mbd5

**Figure 3.**
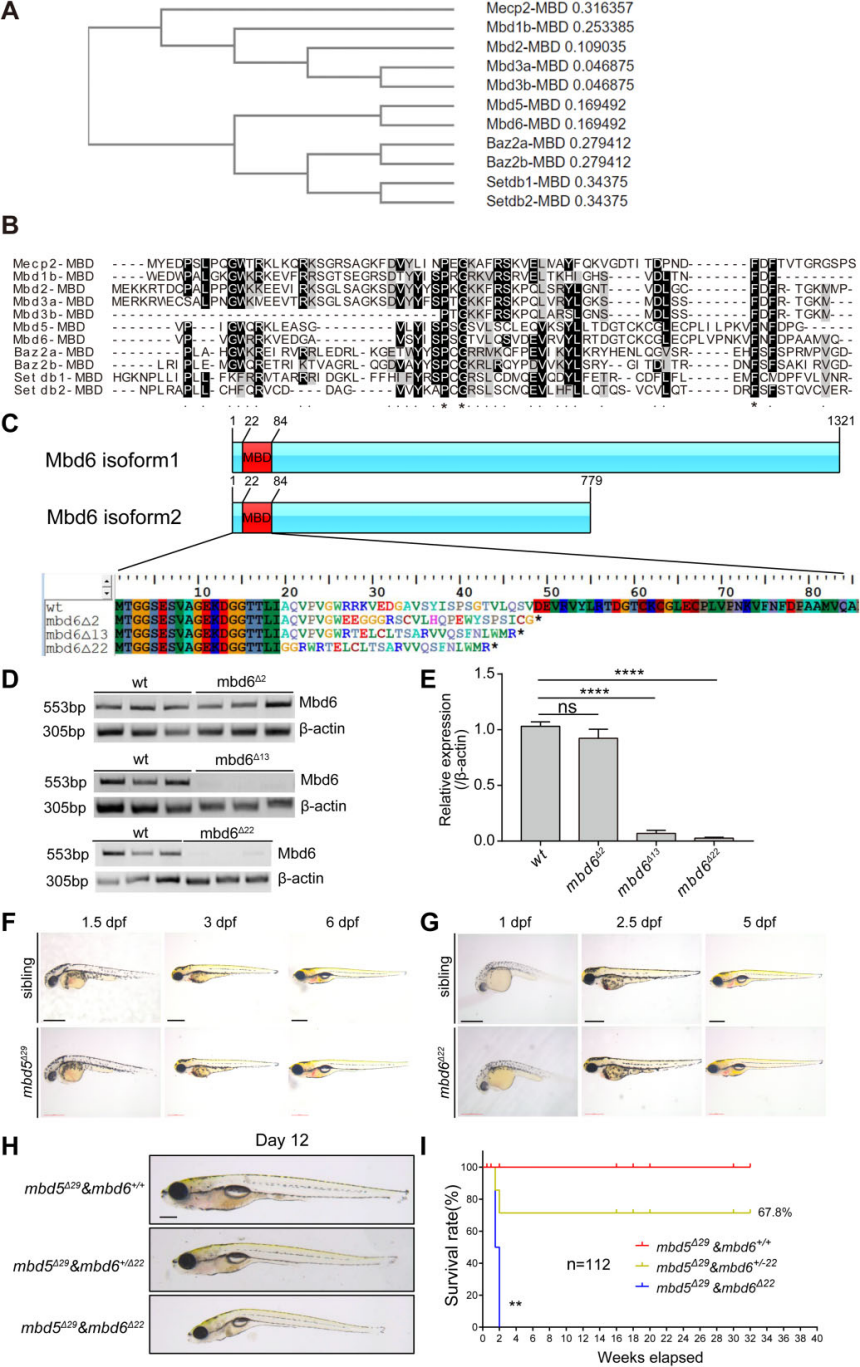
CRISPR/Cas9-mediated germ-line disruption of Mbd6, a MBD protein closely related to Mbd5, reveals redundant function in larval growth and physiology. (**A**) Phylogenetic analysis of the MBD domain-containing proteins. (**B**) Protein alignment of the MBD domain. Asterisks represent identical amino acids among the members. Periods indicate that the amino acid is identical or similar across the members. (**C**) Schematic analysis of the protein variants of Mbd6 and predicted amino acid sequence of wild type and different alleles of *mbd6* mutants. (**D**) Semi-quantitative RT-PCR analysis reveals the *mbd6* mRNA expression in *mbd6*^Δ2^, *mbd6*^Δ13^ and *mbd6*^Δ22^ alleles. β-actin was used as the internal control. (**E**) Statistical analysis of the *mbd6* mRNA expression in *mbd6*^Δ2^, *mbd6*^Δ13^ and *mbd6*^Δ22^ alleles. Value in each column represents mean ± SD, ns, no significance, ****P< 0.0001. (**F**, **G**) Whole-mount lateral view of *mbd5*^Δ29^ and *mbd6*^Δ22^ mutants at indicated developmental stages under bright field condition. At least 10 were examined for each condition. (**H**) Lateral view of *mbd5*^Δ29^& *mbd6*^Δ22^ double mutants and those siblings at 12 dpf. *N* = 48 (*mbd5*^Δ29^& *mbd6^+/^*^+^, *N* = 12; *mbd5*^Δ29^& *mbd6^+/^*^Δ22^, *N* = 28; *mbd5*^Δ29^& *mbd6*^Δ22^, *N* = 8). (**I**) Survival analysis of the progeny of *mbd5*^ΔMBD^& *mbd6^+/^*^Δ22^ in cross during 32 weeks after birth. *N* = 112, Log-rank (Mantel–Cox) test, ** *P*< 0.01. Scale bar: 0.5 mm (F, G).

Our phylogenetic analysis of MBD domain-containing proteins (Figure 3A, B) showed that, among MBD family members, Mbd5 and Mbd6 shared extensive similarity in their MBD domains, which were distinct from other MBD proteins. To determine whether *mbd6* compensates for the loss of *mbd5*, we generated homozygous *mbd6* mutants via CRISPR/Cas9 genome editing. Three mutant alleles were obtained, which yielded predicted truncated proteins of 48 aa, 46 aa and 43 aa, respectively (Figure 3C). Semi-quantitative RT-PCR analysis revealed a significant loss of *mbd6* mRNA in *mbd6*^Δ13^ and *mbd6*^Δ22^ alleles but not in *mbd6*^Δ2^ (Figure 3D, E). Like in the homozygous *mbd5* mutants, no gross morphological defects were observed in *mbd6*^Δ22^ mutants (Figure 3F,G), but the double homozygous mutants (*mbd5*^Δ29^ & *mbd6*^Δ22^) showed reduced whole-body size, displayed ventral curvature at 12 dpf (Figure 3H), and died within two weeks after birth (Figure 3I). Together, these results indicate that *mbd6* can partially compensate for the loss of *mbd5* in zebrafish.

### Alterations in iron metabolism contribute to behavioral abnormalities in the *mbd5* germline KO mutants

Iron, as one of the most abundant elements in the universe, is required to maintain adequate erythropoiesis and sustain physiological activity in vertebrates. Iron deficiency and iron overload both have adverse effects, as evidenced by iron deficiency anemia and inherited hemochromatosis disorder respectively, highlighting the importance of cellular and systematic iron homeostasis (38,39). *Mbd5* KO mice exhibited excessive iron deposition in the liver and iron homeostasis was disrupted in the circulatory system; the expression of intestinal ferritin heavy chain 1 (*Fth1*), a carrier protein that stores iron in hepatocytes and intestinal cells, was reduced in the KO mice (12).

**Figure 4.**
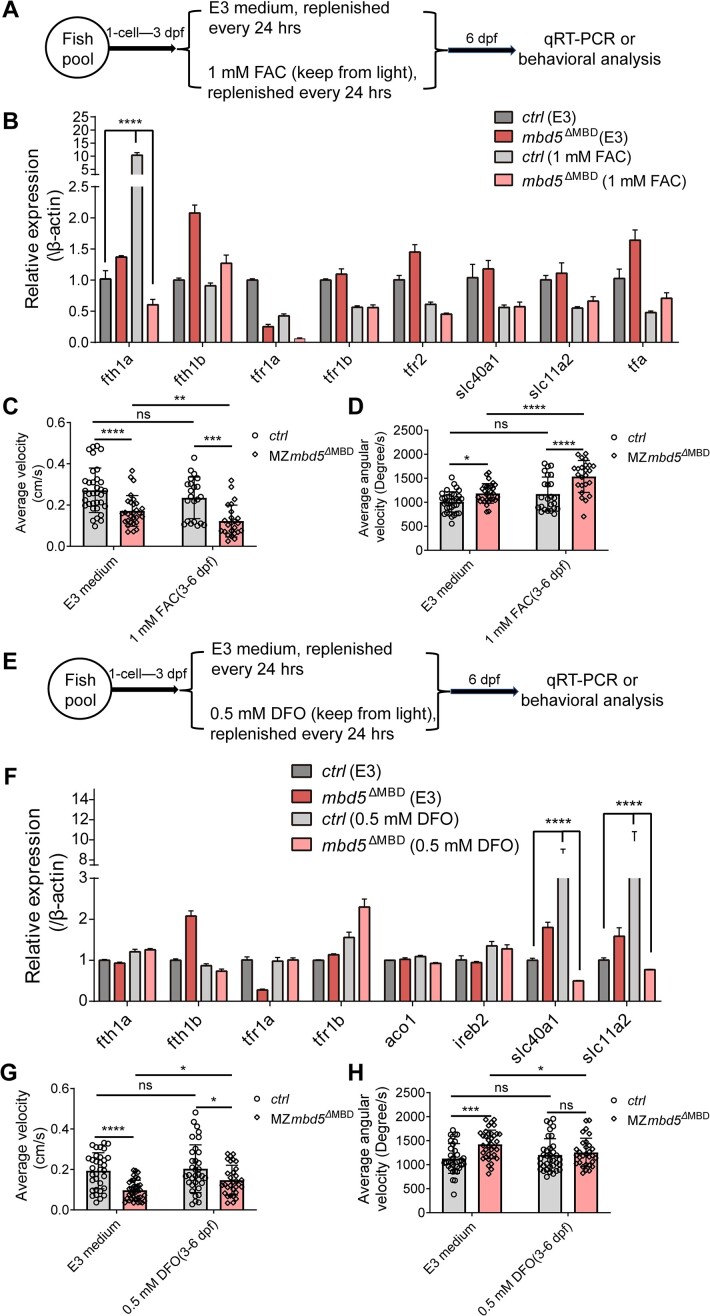
Alterations in iron metabolism contribute to behavioral abnormalities in the *mbd5* germline KO mutant. (**A**) Schematic of FAC treatment prior to gene expression or behavioral analysis. (**B**) Quantitative RT-PCR analysis of iron metabolism-associated genes in *mbd5*^ΔMBD^ mutants upon FAC treatment. (C, D) Scatter plots of average velocity (**C**) and average angular velocity (**D**) in *mbd5*^ΔMBD^ mutants treated with FAC. (**E**) Schematic of DFO treatment. (**F**) Quantitative RT-PCR analysis of iron metabolism-associated genes in *mbd5*^ΔMBD^ mutants upon DFO treatment. (G, H) Scatter plots of average velocity (**G**) and average angular velocity (**H**) in *mbd5*^ΔMBD^ treated with DFO. All data in (C, D, G, H) represent mean ± SD, *N* = 36, two-way ANOVA followed by Dunnett's multiple comparisons test. ns, no significance, *P< 0.05, **P< 0.01, ***P< 0.001, ****P< 0.0001.

We investigated whether iron metabolism was altered, and moreover, whether it contributed to behavioral abnormalities observed in *mbd5* KO zebrafish (Figure 4A). By qRT-PCR analysis, we found that treatment with Ferric ammonium citrate (FAC, a common food-grade supplement for ferric ions) activated the expression of *fth1a* (but not *fth1b*) in a *mbd5*-dependent manner (Figure 4B). Behavioral analysis uncovered that treatment with FAC exacerbated locomotor abnormalities in the MZ*mbd5*^ΔMBD^ mutants (Figure 4C, D).

We next performed the converse experiment, by treating zebrafish with deferoxamine methanesulfonate salt (DFO), an iron chelator, from 3 dpf to 6 dpf (Figure 4E), followed by qRT-PCR and behavioral analysis. DFO treatment activated the expression of solute carrier family 40 member 1 (*slc40a1*) and solute carrier family 11 member 2 (*slc11a2*) in a *mbd5*-dependent manner (Figure 4F). Slc40a1 and Slc11a2 mediate intracellular transport and excretion of iron in most tissues and organs (39,40). Interestingly, behavioral abnormalities in the *mbd5*^ΔMBD^ mutants were partially rescued by DFO treatment, as evidenced by restoration of average velocity and angular velocity parameters closer to wildtype conditions (Figure 4G, H). Together, these results suggest that Mbd5 is essential for activating specific iron metabolism-related genes in response to exogenous signals; alterations in iron metabolism, specifically, iron overload, contribute to behavioral defects observed in the *mbd5*^ΔMBD^ mutants.

### Stage-specific transcriptomic analyses uncover a critical role of Mbd5 in activating genes involved in erythrocyte differentiation and synaptic development

**Figure 5.**
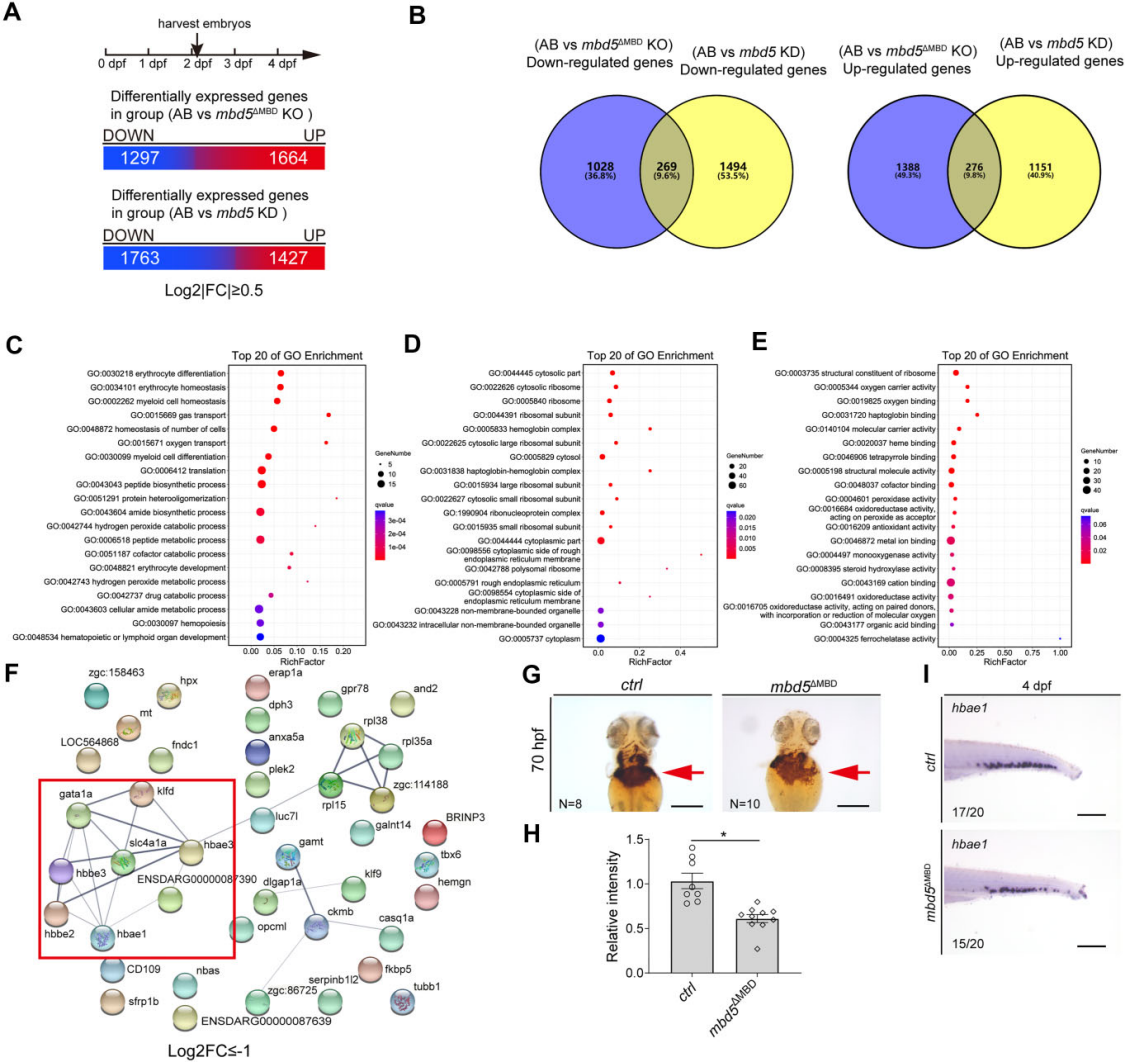
RNA-seq analyses uncover significantly down-regulated biological processes and pathways and highlight deficiency of erythroid differentiation common in both *mbd5*^ΔMBD^ KO and *mbd5* KD embryos. (**A**) Schematic of staged embryo collection and resulted differentially expressed genes in *mbd5*^ΔMBD^ KO and *mbd5* KD samples at 52 hpf at the indicated threshold. (**B**) Venn diagrams of genes downregulated (left) and up-regulated (right) in KO and KD compared to AB WT. (C–E) Gene ontology (GO) analysis of commonly downregulated genes shown in (B, left) reveals significantly altered biological processes (**C**), cellular components (**D**) and molecular functions (**E**) upon disruption of *mbd5*. (**F**) Protein-associated networks analysis (https://string-db.org/) of commonly downregulated genes shown in (B, left) highlights a deficit in erythroid differentiation. (**G**, **H**) O-Dianisidine staining of hemoglobin at 70 hpf and quantification of relative staining intensity. *P*= 0.035, Student's *t*-test. (**I**) Images of *hbae1 in situ* show reduced expression in *mbd5*^ΔMBD^ KO larvae. Scale bar, 200 μm (G, I).

To further dissect the molecular function of *mbd5* in zebrafish development, we carried out transcriptomic analyses at multiple developmental stages. We first analyzed two-day old embryos (∼52 hpf) from *mbd5*^ΔMBD^ germline knockout (KO) and *mbd5* 5′ UTR acute knockdown morphants (KD). All genes whose expression were altered in these Mbd5-deficient fish are potentially important for the phenotypes manifested in them. These genes can be further grouped in two categories: (i) those that represent the primary targets of Mbd5 and (ii) those that are secondary. We reasoned that secondary targets are likely to be linked to different phenotypic manifestations in KD versus KO. Therefore, by looking for overlaps, we removed the likely secondary targets and focused on the plausible primary targets of Mbd5. All data have been subjected to statistical evaluation and thresholding to mitigate noise. 2961 and 3190 genes were differentially expressed in *mbd5*^ΔMBD^ KO and *mbd5* KD embryos compared to sibling controls respectively (log_2_|FC| ≥ 0.5, *P* < 0.05) (Figure 5A). Among them, 269 genes were commonly downregulated, and 276 genes were commonly upregulated (Figure 5B). Gene Ontology (GO) analysis of down-regulated genes uncovered significant changes in biological process (Figure 5C), cellular component (Figure 5D) and molecular function (Figure 5E). STRING analysis at the set threshold also identified significantly altered protein networks (Figure 5F). Among them, processes related to erythrocyte development, oxygen transport, and heme binding prominently stood out. We further validated a significant decrease of erythroid differentiation in *mbd5*^ΔMBD^ mutants by O-Dianisidine staining (Figure 5G, H) and wholemount *in situ* of *hbae1*, one of the most-abundant hemoglobin subunits (Figure 5I).

GO analysis of upregulated genes, however, revealed no significant changes in biological process, cellular component and molecular function, and no functionally associated networks were uncovered with STRING analysis. These results establish that Mbd5 is required to activate gene expression in definable biological processes, cellular components, and molecular function; Mbd5 also represses gene expression, but these repressed genes do not appear to belong to any defined pathways.

Disruption of synaptic development and maturation is a critical contributing factor to ASD (41,42). To study the underlying molecular connections between Mbd5 and ASD, a phenotype observed in humans carrying *MBD5* mutations (7,43), we sought to analyze 7 dpf larval zebrafish, a stage when synaptic development and maturation occur. To uncover not only necessity but also sufficiency of Mbd5 in gene regulation, we established two transgenic lines, *Tg[zhsp70l:FLAGmbd5^iso1^-E2AGFP]* and *Tg[zhsp70l:FLAGmbd5^iso2^-E2AGFP]*, which carried a core cassette containing the heat shock protein 70 (*hsp70*) promoter-driven 3 × Flag fused *mbd5*-E2A-EGFP flanked by Tol2 transposable elements (Supplemental Figure S2A). These transgenic lines also provided an opportunity for *in vivo* labeling of Mbd5 and subsequent biochemical studies, since our custom generated Mbd5 antibody was not suitable for *in vivo* applications (e.g. immunofluorescent labeling or immunoprecipitation). As shown in Figure 1, injection of Mdb5 mRNA rescued the morphant phenotype and did not lead to any apparent gain-of-function phenotypes. Mbd5 transgenic larvae were morphologically indistinguishable from Sib controls after heat shock, despite that altered gene transcription was detected. The Fragments Per Kilobase of transcript per Million mapped reads (FPKM) of Mbd5 transcripts is 5.635 in the Mbd5 transgenic larvae and 4.184 in sib controls upon heat shock, a ratio of Mbd5 expression of 1.34. Thus, there is no overt over-expression of Mbd5 in the transgenic larvae.

Confocal images of germ ring-stage and 48 hpf transgenic embryos showed that both Mbd5^iso2^ and Mbd5^iso1^ were localized to the nucleus. However, Mbd5^iso2^ was largely excluded from the condensed and possibly heterochromatin regions, whereas Mbd5^iso1^ was localized to punctate aggregates inside the nucleus (Supplemental Figure S2B). As shown earlier, Mbd5^iso2^ but not Mbd5^iso1^ mRNA was able to rescue the *mbd5* morphant. Consistent with the observed gain-of-function effect of Mbd5^iso1^ mRNA, we were not able to maintain the Mbd5^iso1^ transgenic line in succeeding generations. We therefore used Mbd5^iso2^ in our studies. Heat-inducible Mbd5^iso2^ expression and self-cleavage of E2A peptide in transgenic animals were validated by western blotting (Supplemental Figure S2C,D).

**Figure 6.**
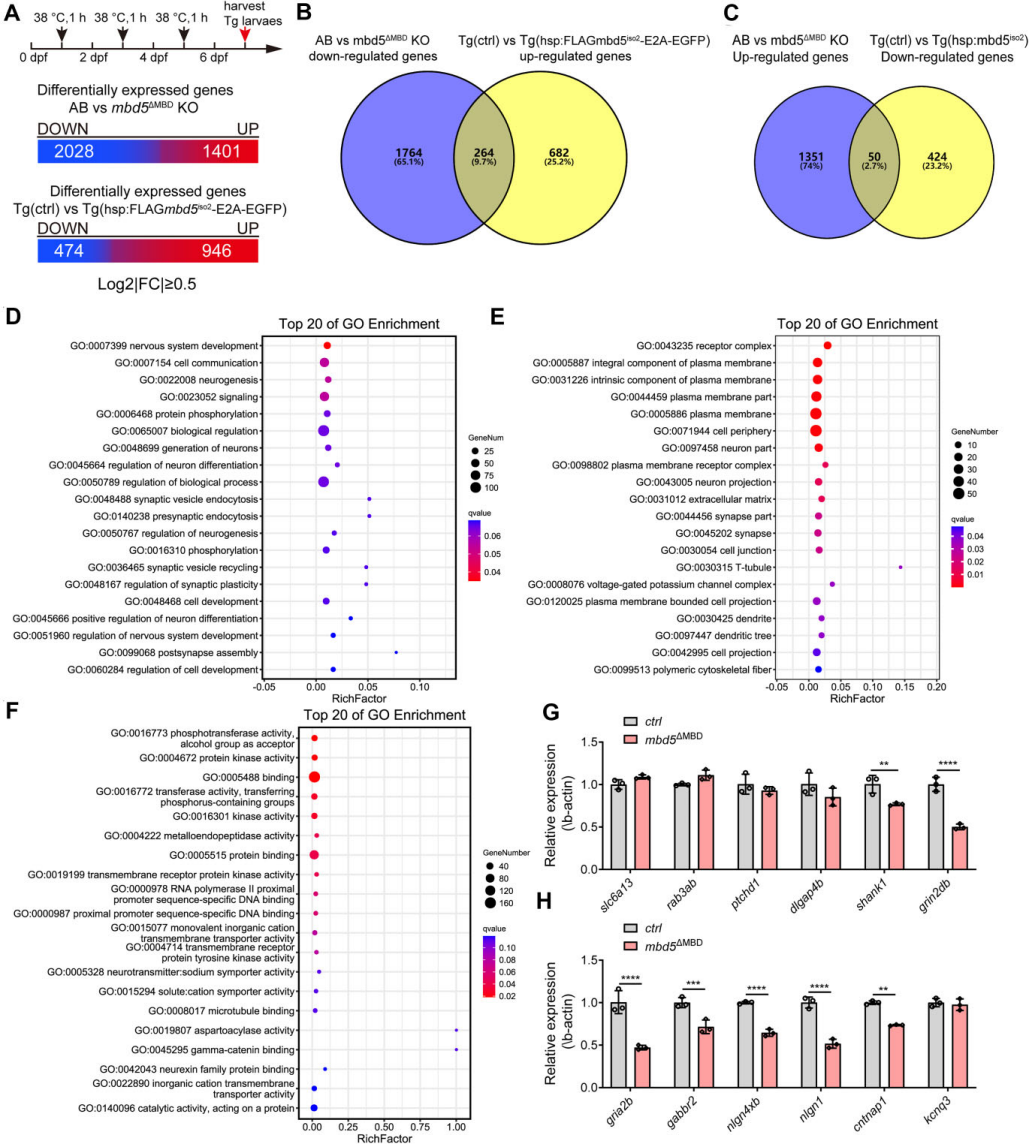
*In vivo* transcriptomic analyses in both *mbd5*^ΔMBD^ KO and *Tg[zhsp70l:FLAGmbd5^iso2^-E2AGFP]* overexpression larval zebrafish reveal a critical role of Mbd5 in activating genes implicated in ASD. (**A**) Schematic of transgenic larval collection and statistical analysis of the differentially expressed genes in *mbd5*^ΔMBD^ KO and *Tg[zhsp70l:FLAGmbd5^iso2^-E2AGFP]* overexpression samples at 7 dpf at the set threshold. (**B**) Venn diagram analysis of down-regulated genes in AB vs *mbd5*^ΔMBD^ KO and up-regulated genes in *Tg(ctrl)* vs *Tg[zhsp70l:FLAGmbd5^iso2^-E2AGFP]*. (**C**) Venn diagram analysis of up-regulated genes in AB vs *mbd5*^ΔMBD^ KO and down-regulated genes in *Tg(ctrl)* vs *Tg[zhsp70l:FLAGmbd5^iso2^-E2AGFP]*. (D–F) GO analysis based on commonly activated genes in (B) reveal significant changes in biological process (**D**), cellular component (**E**) and molecular function (**F**). (G, H) Expression level of genes associated with synaptic development (**G**) and signaling (**H**) were validated through qRT-PCR analysis in *mbd5*^ΔMBD^ mutants. Each value in (G, H) represents mean ± SD, **P< 0.01, ***P< 0.001, ****P< 0.0001.

Transcriptomic analysis of *Tg[zhsp70l:FLAGmbd5^iso2^-E2AGFP]* and *mbd5*^ΔMBD^ KO mutants at 7 dpf uncovered 3429 and 1420 genes that were differentially expressed in *mbd5*^ΔMBD^ KO and transgenic animals compared to control wildtype respectively (log_2_|FC| ≥ 0.5, *P* value < 0.05) (Figure 6A). Among the 2028 down-regulated genes in *mbd5*^ΔMBD^ KO and 946 up-regulated genes in *Tg[zhsp70l:FLAGmbd5^iso2^-E2AGFP]*, 264 genes were shared in common, suggesting that Mbd5 is necessary and sufficient to activate these genes (Figure 6B). Among the 1401 up-regulated genes in *mbd5*^ΔMBD^ KO and 474 down-regulated genes in *Tg[zhsp70l:FLAGmbd5^iso2^-E2AGFP]*, 50 genes were shared in common,, suggesting that Mbd5 is necessary and sufficient to repress these genes (Figure 6C). GO analysis of these commonly affected genes revealed significant changes in biological processes (Figure 6D), cellular components (Figure 6E), and molecular functions (Figure 6F). What stood out prominently were the processes associated with nervous system development and regulation of synaptic plasticity. The expression of genes associated with synaptic development and signaling (e.g*. shank1, gria2b, nlgn1, cntnap1*) were further validated through qRT-PCR analysis in *mbd5*^ΔMBD^ mutants (Figure 6G, H). Together, our transcriptomic analyses show that the expression of the synaptic development/signaling-associated genes implicated in ASD is down-regulated in Mbd5 KO and is up-regulated in Mbd5-overexpressing conditions, supporting the notion that Mbd5 is necessary and sufficient to activate these genes.

### Mbd5 preferentially binds to m^5^C modified mRNAs *in vivo*

**Figure 7.**
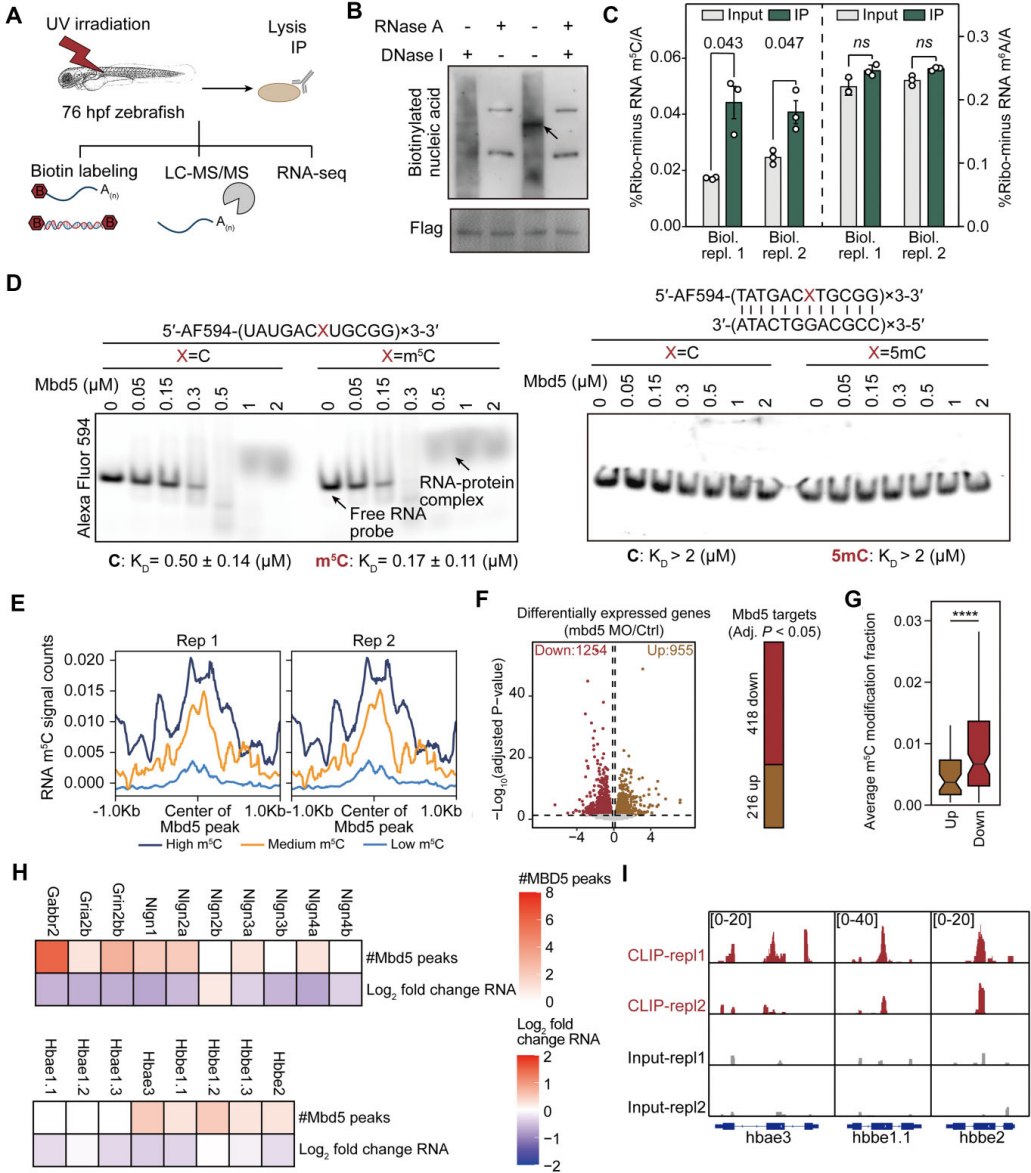
Mbd5 preferentially binds to m^5^C-modified mRNAs and regulates target transcript abundance. (**A**) Schematics showing the analyses of nucleic acids crosslinked to Mbd5 by UV irradiation. (**B**) Immunoblotting showing the detection of biotinylated nucleic acids crosslinked to Mbd5 upon RNase or DNase treatments. The arrow points to the Mbd5 protein band. The two bands detected in the RNase A-treated and RNase A- & DNase I-treated samples represent endogenous biotinylated proteins. (**C**) Quantification of m^5^C abundance in immunoprecipitated Mbd5-bound RNA. As a control, Mbd5 does not enrich m^6^A-modified RNA. (**D**) EMSA assay analyzing binding preference of purified recombinant Mbd5-GFP to RNA oligos (top) or dsDNA (bottom) end-labeled with Alexa Fluor 594 dye. (**E**) Enrichment of RNA m^5^C signal on Mbd5 binding sites. Density plots were generated by plotHeatmap function provided by deeptools package, using the Mbd5 CLIP-seq bigWig files and bed files from the published RNA m^5^C bisulfite sequencing (44). (**F**) Scatter plot showing the differentially expressed genes (left) and Mbd5 direct targets (right) upon *mbd5* MO treatment in zebrafish larvae (76 hpf). P values were determined by DESeq2 algorithm with a Wald test and were corrected for multiple testing using the Benjamini and Hochberg method. (**G**) Boxplot showing the average m^5^C modification fractions of downregulated (Down) or upregulated (Up) genes in the 76 hpf morphants. **** *P* < 0.0001. Student's *t*-test. (**H**) Heatmaps showing the number of Mbd5 binding peaks revealed by CLIP-seq (#Mbd5 peaks) and changes in RNA abundance upon *mbd5* MO treatment (Log2 fold change of RNA) on selective genes important in autism and erythrocyte development. (**I**) IGV tracks showing the binding of Mbd5 to selective genes (*hbae3, hbbe1.1, hbbe2*).

Our results thus far have established a critical role of Mbd5 in embryonic development, iron metabolism, and behavior, through regulating the genes involved in erythrocyte differentiation, iron homeostasis and synaptic development and signaling. To determine the underlying molecular and biochemical mechanisms, we first set out to uncover the physiological nucleic acid substrates of zebrafish Mbd5. Because an IP quality Mbd5 antibody is not available, we used the *Tg[zhsp70l:FLAGmbd5^iso2^-E2AGFP]* zebrafish to perform UV cross-linking and immunoprecipitation (CLIP) with Flag-MBD5 in 76 hpf larval zebrafish that undergo brain development and organogenesis, which are phenotypes of interest in this study. The co-purified nucleic acids were labeled with biotin and visualized by enhanced chemiluminescence (Figure 7A). DNA and RNA were digested with DNase I or RNase A, respectively. We found that RNase treatment completely diminished biotin signals, while DNase treatment resulted in only a moderate decrease of total intensity (Figure 7B). As the nucleic acids, including those co-migrated with Mbd5 (pointed by arrows) and those not (appearing as a dark smear background), completely disappeared in the RNase A-treated sample (the two bands in the RNase A-treated sample are endogenous biotinylated proteins), we conclude that RNase A treatment eliminates the biotin signals from immunoprecipitated nucleic acids. In the DNase I treated sample, a considerable amount of biotinylated nucleic acids was still present, including those co-migrated with Mbd5, albeit fainter. Such reduced signal that co-migrated with Mbd5 in the DNase 1-treated lane could suggest that there was some indirect DNA association with Mbd5, possibly bridged by RNA (see Figure 7D below for a lack of direct DNA binding of Mbd5). Together, this data suggests that, unlike other known MBD proteins that bind to methylated CpG DNA, Mbd5 primarily binds to RNA *in vivo* in zebrafish.

By comparing m^5^C or m^6^A nucleoside abundance to adenosine (A) in both the input and Mbd5-bound RNAs, we found that the RNAs crosslinked to Mbd5 were preferentially enriched for the m^5^C but not m^6^A nucleoside (Figure 7C), therefore, m^6^A was not pursued further in this study. We further validated that purified recombinant Mbd5 directly binds to RNA oligos *in vitro* using an electrophoretic mobility shift assay (EMSA). Methylation of cytosine in the probe promoted Mbd5 binding (0.17 ± 0.11 μM *K*_d_ for the methylated probe compared to 0.50 ± 0.14 μM *K*_d_ for the non-methylated probe) (Figure 7D, left). Zebrafish Mbd5 did not bind to DNA nor 5mC DNA *in vitro* (Figure 7D, right).

We next used CLIP-seq to uncover the RNAs bound by Mbd5 *in vivo*. RNA covalently linked with Mbd5 was immunoprecipitated from 76 hpf larval zebrafish (Supplemental Figure S3A) and identified by short-read RNA sequencing. Mbd5-bound RNAs (a total of 634) were mapped to exons, introns, and intergenic regions. After normalization to nucleotide lengths, an enrichment of Mbd5 peaks was observed in exons, suggesting that Mbd5 preferentially binds to mRNAs (Supplemental Figure S3B). Aligning our CLIP-seq-identified Mbd5 RNA-binding sites with previously mapped RNA m^5^C sites in zebrafish (44) revealed that the peak of Mbd5 binding coincided well with RNA m^5^C sites, and moreover, the higher the m^5^C, the stronger the enrichment of Mbd5 binding (Figure 7E). Together, these data suggest that Mbd5 directly binds to m^5^C-modified RNAs *in vivo* in zebrafish.

To determine how Mbd5 target gene expression might be affected by Mbd5, we performed another RNA-seq at the same stage as that of the CLIP-seq (i.e. 76 hpf) using control and *mbd5* morphants, which uncovered 1254 and 955 genes that are down- or up-regulated respectively in *mbd5* morphants. By aligning this RNA-seq dataset with and the CLIP-seq dataset, we found that among the Mbd5 direct target genes, 418 were down-regulated, and 216 were up-regulated in *mbd5* morphants (Figure 7F). Intriguingly, down-regulated genes showed significantly more RNA m^5^C modifications than up-regulated genes (Figure 7G). Mbd5 mRNA binding sites showed an enriched C-rich RNA motif (Supplemental Figure S3C). The genes downregulated in the *mbd5* morphants are enriched for GO terms such as synaptic signaling and inorganic cation transmembrane transport (Supplemental Figure S3D). mRNAs encoded by genes related to synaptic formation and ASD (e.g. certain *nlgns*, *grin2bb, gria2b, and gabbr2*) and genes critical for erythrocyte differentiation (e.g. *hbbe2, hbbe1, and hbae3*) were bound by Mbd5 and were downregulated after Mbd5 knockdown (Figure 7H). Example IGV tracks from the CLIP-seq were shown in Figure 7I, and we further validated the binding of Mbd5 to *hbae3*, *hbae1.1*, *hbbe2, gabbr2, grin2bb* and *nlgn1* with CLIP-qPCR (Supplemental Figure S3E). Taken together, direct binding of Mbd5 to its target mRNAs regulates target gene expression *in vivo*: While Mbd5 promotes the expression of most target genes (418 at 76 hpf), it also decreases the levels of a subset of them (216 at 76 hpf).

### Mbd5 interacts with the PR-DUB complex to promote histone H2A deubiquitylation *in vivo*

**Figure 8.**
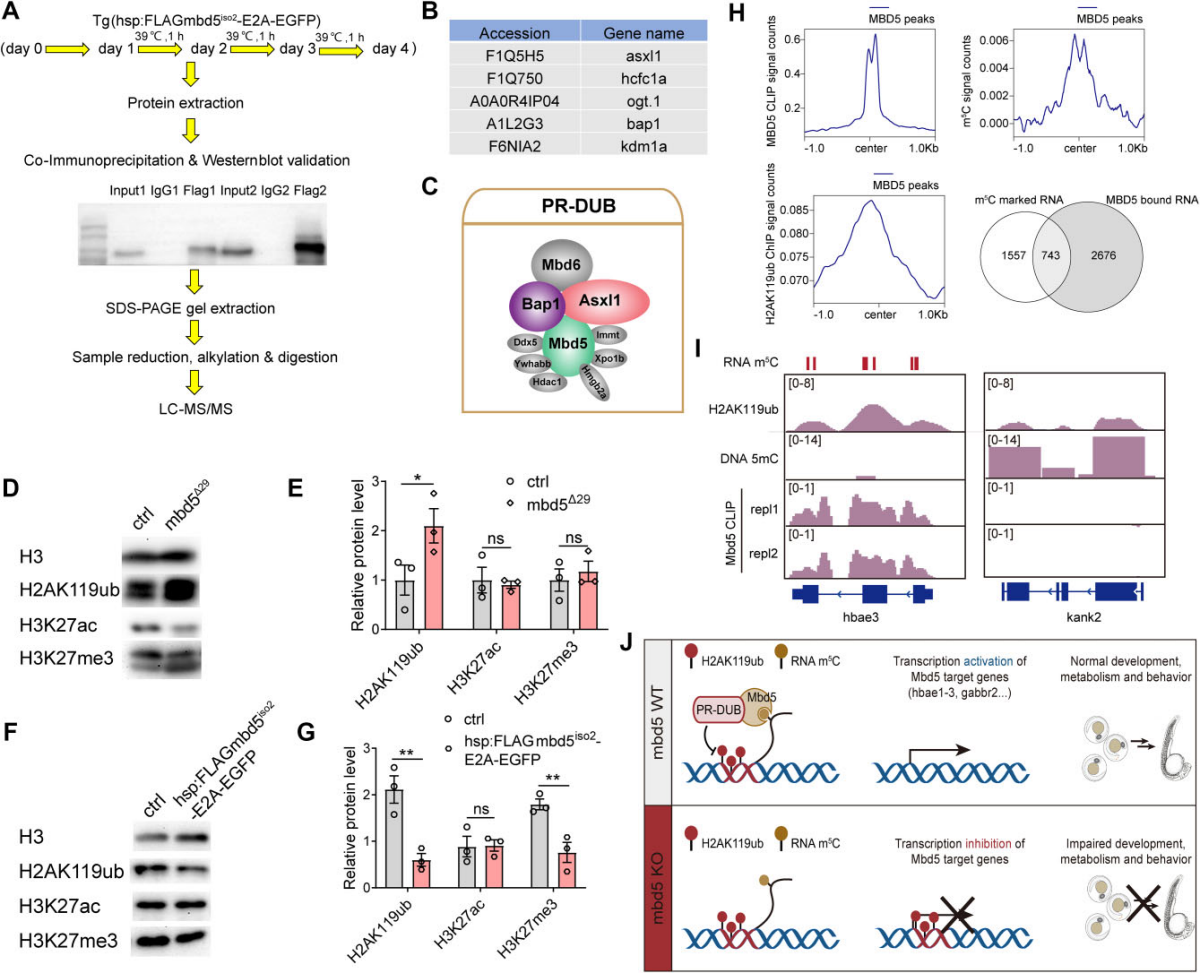
Mbd5 interacts with PR-DUB and is necessary and sufficient to promote deubiquitylation. (**A**) Schematic of the experimental flow of *in vivo* co-IP and mass spectrometry. (**B**) A table listing all proteins belonging to the PR-DUB complex identified in LC–MS/MS. (**C**) Schematic diagram of the PR-DUB complex. (**D**–**G**) Representative images of western-blot and quantification of the expression level of H2AK119ub1, H3K27ac and H3K27me3 in *mbd5*^Δ29^ mutants (D, E), and in *Tg[zhsp70l:FLAGmbd5^iso2^-E2AGFP]* (F-G). H3 was used as the internal control. All data in E and G represent mean ± SEM, ns, no significance, *P< 0.05, **P< 0.01. (**H**) Enrichment of the Mbd5 CLIP signal (top left), RNA m^5^C signal (top right), and histone H2AK119ub1 (bottom left) on Mbd5 targets. The Venn diagram depicts the overlap between m^5^C-marked RNAs and Mbd5-bound RNAs. The density plot (top right) was generated by plotHeatmap function provided by deeptools package, using the Mbd5 CLIP-seq bigWig files and bed files from the published RNA m^5^C bisulfite sequencing (44). The density plot (bottom left) was generated by plotHeatmap function provided by deeptools package, using the Mbd5 CLIP-seq bigWig files and bed files from H2AK119ub1 ChIP-seq datasets (52). (**I**) IGV tracks showing DNA 5mC, RNA m^5^C, and H2AK119ub1 occupancy on an example Mbd5 target (*hbae3*) and an example non-Mbd5 target (*kank2*). (**J**) A model: the zebrafish Mbd5 protein is a mRNA m^5^C reader. By binding to nascent m^5^C-modified mRNA, it recruits or stabilizes the PR-DUB complex to promote histone deubiquitylation at H2AK119 sites. Mbd5 thus regulates the transcription of genes required for normal development, metabolism, and behavior.

We next sought to understand how the binding of Mbd5 to m^5^C mRNAs regulates target gene expression. Using the *Tg[zhsp70l:FLAGmbd5^iso2^-E2AGFP]* zebrafish, we performed heat shock at multiple developmental stages and carried out co-immunoprecipitation (co-IP) at 4 dpf using the FLAG antibody, followed by mass spectrometry (Figure 8A). Proteins with significant enrichment in three rounds of co-IP/Mass Spec experiments were identified (Supplemental Table S5 and S6). One protein, Asxl1, caught our attention, as it is a core component of the PR-DUB complex (Figure 8B,C), which has been previously shown to interact with human MBD5 in Hela cells (13). The PR-DUB complex, including both the core components (Asxl1 and Bap1) and associated proteins, removes the repressive chromatin mark monoubiquitin from histone H2A (H2AK119ub) (45). The observations that Asxl1 were co-IPed with zfMbd5 in all three rounds whereas other PR-DUB components were co-IPed with zf Mbd5 in at least one round of co-IP suggested that Mbd5 bound more tightly with Asxl1 than with other PR-DUB complex components. Another noteworthy protein complex co-IPed with zfMbd5 is the non-canonical (variant) PRC1 complex components HDAC1 (detected in all three rounds of co-IP) and CBX3a (detected in one round of co-IP). PRC1 is involved in H2A ubiquitylation (46). Given that PR-DUB interacts with both human and zebrafish MBD5, we chose to focus on PR-DUB in this study.

To determine which domains of Mbd5 interact with PR-DUB, we dissected the structure-function relationship of Mbd5 interaction with Asxl1 and Bap1 in HEK293 cells. Asxl1 and Bap1 are the core components of the PR-DUB complex and were most consistently identified in all four rounds of our co-IP and LC/MS/MS experiments. Two constructs of EGFP-fused MBD domain and EGFP-fused PWWP domain were generated (Supplemental Figure S4A). After verifying that the full-length zebrafish Mbd5 isoform 2 co-IPed with zebrafish Asxl1 (Supplemental Figure S4B), we co-transfected EGFP-fused MBD domain or EGFP-fused PWWP domain of Mbd5 with myc-tagged Asxl1. The MBD domain was sufficient to interact with Asxl1 (Supplemental Figure S4C), and very weak interaction was revealed between the PWWP domain and Asxl1 (Supplemental Figure S4D). The MBD domain was also found to interact with Bap1 (Supplemental Figure S4E). Moreover, we found that human MBD5^iso2^ and human ASXL1(Y591X), a form that is competent for activating PR-DUB (31), mutually enhanced the protein stability when co-transfected in HEK293T cells (Supplemental Figure S4F, compare the middle lane to the left or right lanes). Together, our unbiased *in vivo* co-IP coupled with mass spectrometry detects a single protein complex PR-DUB with which Mbd5 interacts. Such interaction is largely between the MBD domain and Asxl1 and Bap1. Our data using human MBD5 and ASXL1 also suggest that MBD5 and PR-DUB interaction serves to stabilize both MBD5 and ASXL1.

As core components of the PR-DUB complex, ASXL1/2/3 and BAP1 regulate epigenetic modification of histones in various tissues and in the context of human cancer (47–50). PR-DUB’s deubiquinase activity erases the histone H2A lysine 119 ubiquitination (H2AK119ub1), a commonly known repressive chromatin mark. This modification in turn could indirectly impact other types of histone modifications (45,51). We therefore wondered whether Mbd5 played a role in modifying the histone epigenetic codes. Western-blot analysis found that H2AK119ub1 was up-regulated in *mbd5*^Δ29^ mutants (Figure 8D, E) and down-regulated in *Tg[zhsp70l:FLAGmbd5^iso2^-E2AGFP]* that over-expressed Mbd5 (Figure 8F, G). Thus, Mbd5 is necessary and sufficient to promote histone H2AK119 deubiquitylation *in vivo*.

Acetylation of lysine at the N-terminal position 27 of histone H3 (H3K27ac) is an activating chromatin mark associated with activation of transcription. H3K27ac was unchanged in both the *mbd5*^Δ29^ mutant (Figure 8D,E) and *Tg[zhsp70l:FLAGmbd5^iso2^-E2AGFP]* Mbd5 over-expressing conditions (Figure 8F, G), suggesting that Mbd5 does not affect this particular histone modification. We also examined H3K27me3, methylation of lysine 27 on histone 3, which is a modification usually associated with gene repression. H3K27me3 was unchanged in the *mbd5*^Δ29^ mutant (Figure 8D, E), but was significantly decreased in *Tg[zhsp70l:FLAGmbd5^iso2^-E2AGFP]* under the Mbd5 overexpressing condition (Figure 8F, G). This observation suggests that Mbd5 is sufficient to decrease H3K27me3; the lack of change in the *mbd5*^Δ29^ mutant is possibly due to redundancy or compensation.

Next, we analyzed genome-wide the Mbd5 CLIP-seq signals in the context of RNA m^5^C sites previously detected with bisulfate sequencing (44) and H2AK119ub1 sites previously mapped via ChIP-seq (52). The center of Mbd5 binding on its target RNAs co-localized with both the RNA m^5^C and the H2AK119ub1 peak signals (Figure 8H). Between the previously discovered RNA m^5^C sites (44) and the Mbd5 RNA interaction sites discovered in this study, an overlap of 743 was detected (Figure 8H). As examples, the signals were shown on one Mbd5 target (*hbae3*) and one non-target (*kank2*) loci (Figure 8I). Taken together, these analyses reveal co-localization of Mbd5 mRNA binding sites with those of RNA m^5^C and H2AK119ub1 marks.

## Discussion

The data presented in this study have led us to propose the following working model: The zebrafish Mbd5 is an mRNA m^5^C reader that interprets m^5^C modification on target mRNAs to recruit and/or stabilize the histone remodeling complex PR-DUB on the target gene chromatin; PR-DUB further removes the histone mark H2AK119ub1. For most of the Mbd5 target loci (418), removal of H2AK119ub1 results in an activation of transcription (Figure 8J). However, we also found that Mbd5 is required to repress the expression of a subset of target genes (216). This observation is consistent with recent findings that suggest a more complex role of PR-DUB in regulating gene expression: Rather than simply turning on or off gene transcription, the removal of H2AK119ub1 serves to reconfigure the chromatin landscape and poise genes for either activation or repression (53,54). Alternatively, the interaction between Mbd5 and the non-canonical (variant) PRC1 complex might underlie the repressive effects of Mbd5 on its target genes. Although the binding sites of Mbd5 on its target RNAs coincide well with both the RNA m^5^C and the H2AK119ub1 peak signals, at present, we do not know whether Mbd5 binding to RNA m^5^C and its interaction with PR-DUB occur concurrently on Mbd5 target genes. Mbd5’s role as an RNA m^5^C reader could be functionally independent from its role as an PR-DUB interactor. Future biochemical studies to determine whether m^5^C modification on RNAs can occur co-transcriptionally prior to their release and whether m^5^C modified RNAs can be co-immunoprecipitated with Mbd5 and PR-DUB complex are needed to further test our model. Additionally, our data show that Mbd6, a protein with the MBD domain closely related to MBD5, can phenotypically compensate for the loss of Mbd5, suggesting that Mbd6 may have similar roles as Mbd5. Moreover, it would be of interest to examine the *in vivo* roles of two major mRNA m^5^C writers, NSun2 and NSun6 (44), in the context of our findings regarding Mbd5.

Advances in reverse genetic technologies such as CRISPR have enabled the generation of germline mutations in a wide variety of organisms including zebrafish (55). They also reveal phenotypic differences between acute KD (e.g. with morpholino antisense oligonucleotides) and germline KO: the latter often have milder phenotypes than the former, due to a phenomenon known as genetic compensation (37). Mutant mRNA degradation has been shown to trigger genetic compensation leading to mild or no phenotypes in germline KOs (56,57). In the case of Mbd5, we observed severe developmental deficits in KDs, whereas KOs survived to adulthood and suffered from defects ranging from erythrocyte differentiation, iron metabolism, to behavior. Such milder phenotypes compared to KDs were observed in multiple germline KO alleles recovered. Intriguingly, in the *mbd5*^ΔMBD^ mutants, which carried an in-frame deletion of the entire MBD domain, the *mbd5* mRNA levels were not noticeably different from WT siblings (Supplemental Figure S1E). This suggests that mechanisms other than mutant mRNA degradation might be at play in mediating genetic compensation.

Mbd5 is an evolutionarily conserved MBD domain-containing protein disrupted in multiple human disorders. By applying a combination of genetic, molecular, biochemical, pharmacological, and behavioral approaches in zebrafish, we show that Mbd5 primarily activates gene expression in defined biological pathways that are critical for embryonic development, erythrocyte differentiation, iron metabolism, and behavior. Mbd5 activity is required to activate synaptic development and synaptic signaling-associated genes implicated in human ASDs. Intriguingly, one of the major mRNA m^5^C writers, NSun2, has recently been associated with ASD and is required for a protein synthesis-dependent form of synaptic plasticity (58). Defects in both iron metabolism and synaptic development/signaling contribute to behavioral dysregulation. The diverse phenotypes observed in both zebrafish and humans are connected by the versatility of Mbd5 in binding to enriched motifs that can be found in many different target RNAs.

Our findings bring the following new advances to the research areas of epigenetics and gene regulation. First, the discovery of zebrafish Mbd5 as a m^5^C RNA reader sheds new light on the versatility of MBD proteins across different family members and evolutionary trees, indicating that they not only interpret DNA but also RNA methylation. Second, our finding of Mbd5 binding to m^5^C mRNA and PR-DUB and facilitation of histone deubiquitylation suggests a tantalizing feedback loop for regulating gene transcription, in the context of vertebrate embryonic development, iron metabolism, and behavior. Finally, RNA m^5^C is previously shown to facilitate zebrafish maternal-to-zygotic transition by stabilizing maternal mRNAs through the reader YBX1 (59). The molecular mechanism by which m^5^C mRNA may be utilized to regulate transcription is unknown. By identifying Mbd5 as an mRNA m^5^C reader and as a regulator of histone epigenetic codes via its interaction with PR-DUB, it raises a tantalizing possibility that m^5^C mRNA may regulate transcription via histone modification. A future direction is to further elucidate how Mbd5 might engage both mRNA m^5^C and the PR-DUB complex on its target genes that result in either transcription activation or repression.

## Supplementary Material

gkae093_Supplemental_File

## Data Availability

The data underlying this article will be shared on reasonable request to the corresponding author. Sequencing data have been deposited into the Gene Expression Omnibus (GEO) under the accession number GSE222803 (token: mbinagiatvkzdsd) and GSE225204 (token: olqboomapxgzvyz) for RNA sequencings. The mass spectrometry proteomics data have been deposited to the ProteomeXchange Consortium via the PRIDE (60) partner repository with the dataset identifier PXD048128.

## Notes

Present address: Xiang Zhao, Chan Zuckerberg Biohub, San Francisco, CA 94158, USA.

Present address: Yimin Wang, GeneMind Biosciences Company Limited, Shenzhen, China.
