# Supplementary material for: Zebrafish Mbd5 binds to RNA m5C and regulates histone deubiquitylation and gene expression in development metabolism and behavior
Source: Nucleic Acids Res. 2024 Feb 15;52(8):4257–75. doi: 10.1093/nar/gkae093 (PMC11077058; doi:10.1093/nar/gkae093)
Supplement: gkae093_Supplemental_File [file gkae093_supplemental_file.pdf]

Supplemental Files

The Supplemental Files for Guo et al include four Supplemental Figures and six Supplemental Tables.

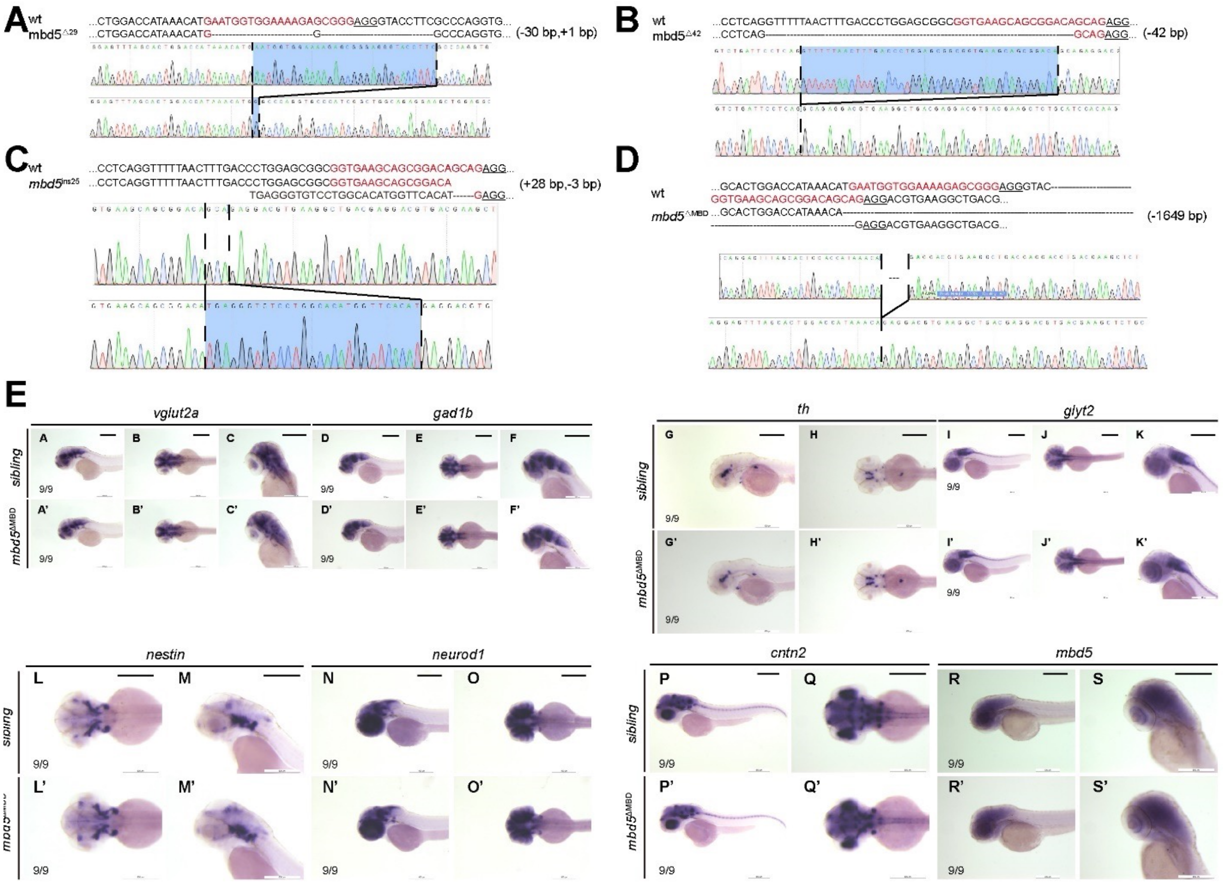

Supplemental Fig. S1: Sanger sequencing validation of CRISPR-induced *mbd5* germline mutants, which express apparently normal neurotransmitter and neurodevelopmental markers. (**A-D**) Sanger sequencing validation of *mbd5*$^{\Delta29}$ mutants (**A**), *mbd5*$^{\Delta42}$ mutants (**B**), *mbd5*^{ins25} mutants (**C**), *mbd5*$^{\Delta MBD}$ mutants (**D**). The bases marked in red represent sgRNA target sites, followed by underlined bases which indicate the PAM motif. (**E**) Wholemount *in situ* images of siblings (**A-S**) and *mbd5*$^{\Delta MBD}$ mutants (**A'-S'**) at 48 hpf. (**A-C'**): vesicular glutamate transporter (*vglut2a*); (**D-F'**): glutamate decarboxylase 1b (*gad1b*); (**G-H'**): tyrosine hydroxylase (*th*); (**I-K'**): solute carrier family 6 (neurotransmitter transporter, glycine), member 5 (*glyt2* or *slc6a5*); (**L-M'**): a member of intermediate filament protein family (*nestin*); (**N-O'**): neuronal differentiation 1 (*neurod1*); (**P-Q'**): transiently expressed axonal glycoprotein (*tag1*) or contactin 2(*cntn2*); (**R-S'**): methyl-CpG binding protein 5 (*mbd5*). No gross differences were observed between the mutant and their sib controls. (**A-A'**, **D-D'**, **F-G'**, **I-I'**, **K-K'**, **M-N'**, **P-P'**, **R-R'**): lateral view; (**B-B'**, **E-E'**, **H-H'**, **J-J'**, **L-L'**, **O-O'**, **Q-Q'**): dorsal view; (**C-C'**, **S-S'**): anticline view. Scale bar: 500 µm.

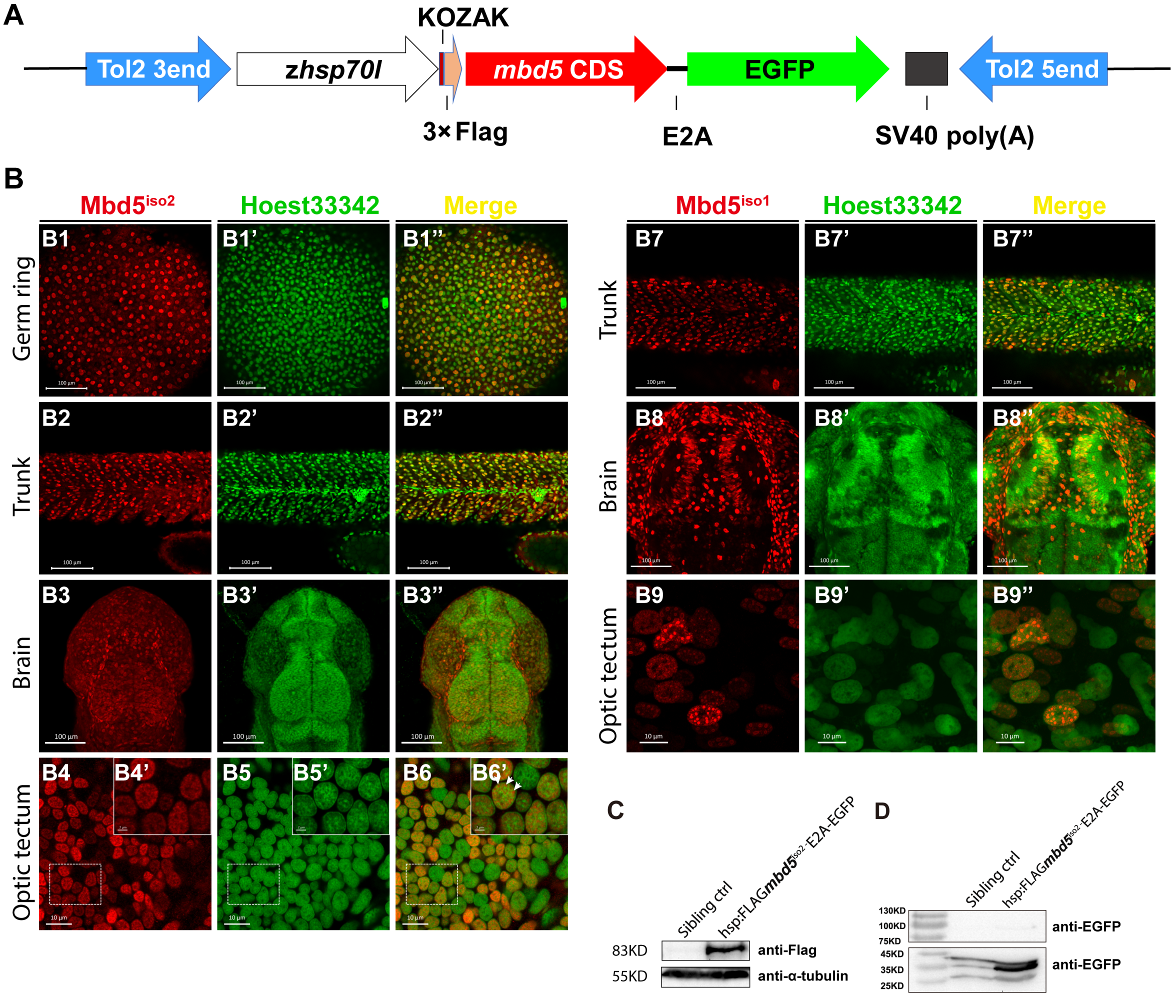

Supplemental Fig. S2. Construction of *mbd5*$^{\Delta MBD}$ KO and *Tg[zhsp70l:FLAGmbd5*^{iso2}*-E2AGFP]* overexpression larval zebrafish models. (**A**) Schematic showing the core cassette of *zhsp70l* promoter-driven 3×Flag fused *mbd5*-E2A-EGFP flanked by Tol2 arms. (**B**) Confocal images of Germ ring-stage embryos (B1-B1”), trunk, brain and optic tectum from whole mount *Tg(zhsp70l:FLAG-mbd5*^{iso2}*-E2A-EGFP)* zebrafish at 48 hpf immuno-stained for Flag (Red, Mbd5^{iso2}) and Hoest33342 (green, DNA)(B2-B6). Confocal images of trunk, brain and optic tectum from whole mount *Tg(zhsp70l:FLAG-mbd5*^{iso1}*-E2A-EGFP)* zebrafish at 48 hpf immuno-stained for Flag (Red, Mbd5^{iso1}) and Hoest33342 (green, DNA)(B7-B9). (**C-D**) Western-blot validation of Mbd5^{iso2} expression and self-cleavage of E2A peptide in *Tg(zhsp70l:FLAG-mbd5*^{iso2}*-E2A-EGFP)* zebrafish. Scale bar: 100 μm (**B1-B3”**, **B7-B8’’**), 10 μm (**B4-B6**, **B9-B9’’**), 2 μm (**B4’-B6’**).

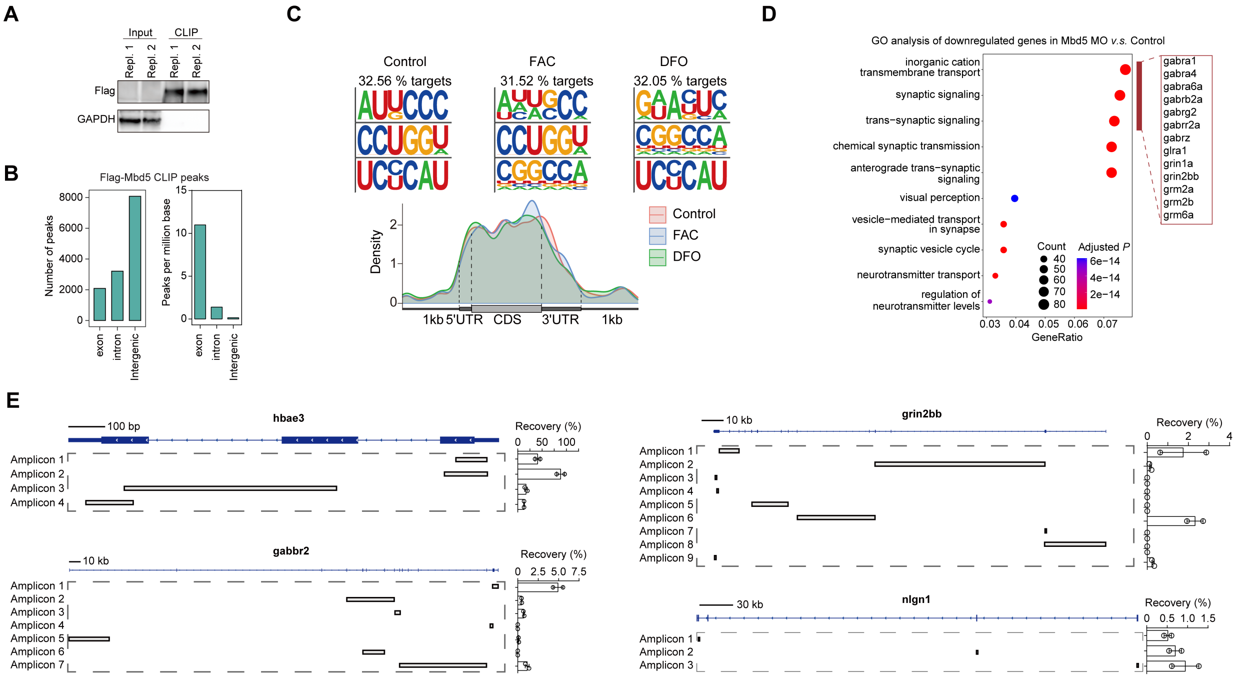

Supplemental Fig. S3. Mbd5 preferentially binds to exonic regions of mRNAs. (**A**) Validation of CLIP-Seq procedure with 76 hpf zebrafish larvae by western blot. (**B**) Distribution of Mbd5 CLIP peaks on RNA. (**C**) Enriched nucleotide motifs (top) and metagene profiles (bottom) of MBD5 as calculated by HOMER. (**D**) Enriched gene ontology (GO) terms (biological pathways) for genes downregulated in 76 hpf *mbd5* morphants. (**E**) Illustrations showing the CLIP fragment-qPCR to assay Mbd5 binding on specific transcripts. Right: individual quantifications for the recovery rate in immunoprecipitation.

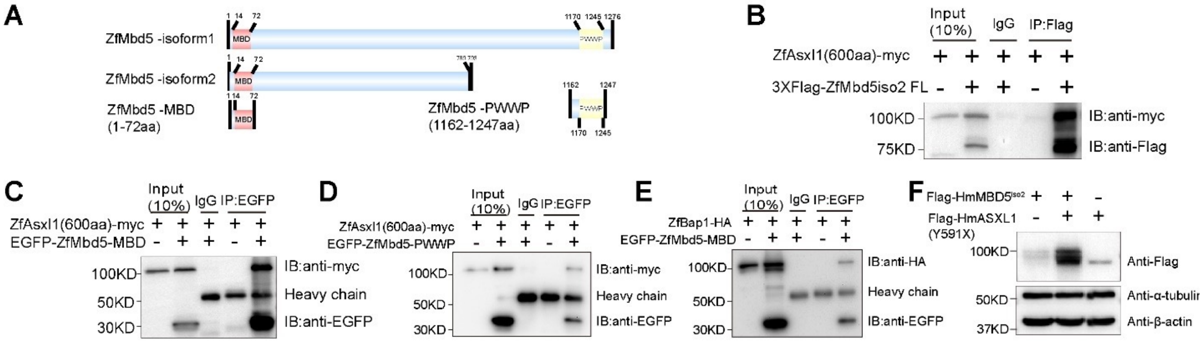

Supplemental Fig. S4. Mbd5 interacts with PR-DUB through its MBD domain and this interaction stabilizes both human ASXL and MBD5. (**A**) Schematic diagram of the protein structure of zebrafish Mbd5, MBD and PWWP domain, which were used for *in vitro* Co-IP validation. (**B-E**) *In vitro* co-IP validation of ZfMbd5^{iso2} FL, ZfMbd5-MBD, ZfMbd5^{iso1}-PWWP with ZfAsxl1(1-600aa), and ZfMbd5-MBD with ZfBap1 in HEK293T cells. (**F**) HEK293T cells were co-transfected with plasmids expressing HmMBD5^{iso2} and HmASXL1(Y591X), showing that co-transfection results in higher levels of both proteins compared to single transfection.

Supplemental Tables S1-S6

Table S1. The sequences of sgRNA target sites and primers used for genotyping

mbd5 E2 sgRNA-Fwd	aattTAATACGACTCACTATAGGGAATGGTGGAAAAGAGCGGGGTTTAAGAGCTATGCT
mbd5 E4 sgRNA-Fwd	aattTAATACGACTCACTATAGGGGTGAAGCAGCGGACAGCAGTTTAAGAGCTATGCT
mbd6 E3 sgRNA-Fwd	aattTAATACGACTCACTATAGGGTCGGCTGGCGGAGGAAGGTTTAAGAGCTATGCT
General sgRNA-Rev	AAAAAAAGCACCGACTCGGTGCCAC
mbd5 E2 GT-F1	TCAGTCTCCACTCTACTTCTTTCGG
mbd5 E2 GT-R1	AGATCAGATCGAGCAGGAGATCCTT
mbd5 E2 GT-F2	CCTCTTCTGAGCGTCAGGATG
mbd5 E2 GT-R2	GATCGCTTTAAAACTAAACTGCATC
mbd5 E4 GT-F1	TGCGCCTGTATGCTGACTCT
mbd5 E4 GT-R1	TGCAGCTCCATGCTCTTATGC
mbd5 E4 GT-F2	GAACTCTGACCCTCAGCTCG
mbd5 E4 GT-R2	CTTGAAGGGGTTCCTGCACT
mbd5$^{\Delta MBD}$ (wt/het)-F	AGAATAACACACGACATTAACCCAG
mbd5$^{\Delta MBD}$ (wt/het)-R	AGAAGCAATCATTGACTAAAGGCTA
mbd5$^{\Delta MBD}$ (het/homo)-F	CTCCACTCTACTTCTTTCGGACTCT
mbd5$^{\Delta MBD}$ (het/homo)-R	TACTGGTGCCTCCTCCGGG
m bd6 E3 SA-F1	TGCGAACCGTTAAATTAGCCTG
m bd6 E3 SA-R1	GAACGTTTGCTCGGGTGAAG
m bd6 E3 SA-F2	GGGGCAGTGAGAGTGTAGCAG
m bd6 E3 SA-R2	TACCTTTATTTAGCAGCAACGGC

Table S2. PCR primers for generating RNA in situ probes

gad1b -Fwd	CGTCTTCTGCACCTTCTTCCT
gad1b -sp6-Rev	AATTATTTAGGTGACACTATAGAAGATGTGAACAGCACGAGCC
vglut2a -Fwd	AGGGAGCCTGCTGGTTTTAG
vglut2a -sp6-Rev	AATTATTTAGGTGACACTATAGACTGCAGGTCCTAGCAGCTTAG
glyt2 -Fwd	TTGTGACGTGTACGAACAGC
glyt2 -sp6-Rev	AATTATTTAGGTGACACTATAGACAAGTGGGTCGATCATGTTCT
neurod1 -Fwd	GCAGGATGCCTCCAACTGA
neurod1 -sp6-Rev	AATTATTTAGGTGACACTATAGAGTGACCGCAACGTAGAAGC
cntn2 -Fwd	GAGATGGAGCCGTGGGTATG
cntn2 -sp6-Rev	AATTATTTAGGTGACACTATAGATGGACCATCATTCCAGTGCC
th -Fwd	ACCAAAGGATGGCTTGGAGG
th -sp6-Rev	AATTATTTAGGTGACACTATAGAGAACCGCACAGAAAACGGTC
nestin -Fwd	GCGAGGAGGTGACAGAACAA
nestin -sp6-Rev	AATTATTTAGGTGACACTATAGAGGCTGCATCTGAAATGCTCG
mbd5 -Fwd	ATGTCCACTCATCCTCCCGA
mbd5 -sp6-Rev	AATTATTTAGGTGACACTATAGATACATGGGGTTGTGCTGACC
mbd5 -T7-Fwd	AATTTAATACGACTCACTATAGGATGTCCACTCATCCTCCCGA
mbd5 -Rev	TACATGGGGTTGTGCTGACC
mbd5 ^{iso1}-Fwd	AGAATAAGATGCAGCTGGCAGA
mbd5 ^{iso1}-sp6-Rev	AATTATTTAGGTGACACTATAGATTGAGTGTGACCGGATGAGC
mbd5 ^{iso1}-T7-Fwd	AATTTAATACGACTCACTATAGGAGAATAAGATGCAGCTGGCAGA
mbd5 ^{iso1}-Rev	TTGAGTGTGACCGGATGAGC
mbd6 -Fwd	CATTCCTGTCCCTCAATCCCG
mbd6 -sp6-Rev	AATTATTTAGGTGACACTATAGACATTGAGACAGACAGC
hbae1 -Fwd	CCAAAGACAAAGCTGCCGTC
hbae1 -T7-Rev	AATTTAATACGACTCACTATAGGCACGAGGATGTTGTGGGACA

Table S3. qRT-PCR primers for validating RNA-seq candidates

fth1a -qRT-Fwd	AGAGGGGAGGGAGGATCTTT
fth1a -qRT-Rev	CACCACTTCCCCACTCGT
fth1b -qRT-Fwd	GGCAATAAACCGCCAGATT
fth1b -qRT-Rev	AGACTTGTCATCCCTGTCGAA
tfr1a -qRT-Fwd	AAAACTGTCTGCGGATTCGT
tfr1a -qRT-Rev	CATGTAACCAATGAGGTATCCAAA
tfr1b -qRT-Fwd	CATCGACCAGGTGCTAAACA
tfr1b -qRT-Rev	ATGTCTCCTGCCAGGTCATT
tfr2 -qRT-Fwd	TTCCCTCGTTTAACCACACC
tfr2 -qRT-Rev	CACTGCACCTGAGAGCTGAC
slc40a1 -qRT-Fwd	CCCAGTAGAGTCCTACATGTCTGTT
slc40a1 -qRT-Rev	GGTCAAGTCGAAGGACCAAA
slc11a2 -qRT-Fwd	GCGGACCACTGCACTTCT
slc11a2 -qRT-Rev	CGTCCCGTCCAGTCTTCA
mbd5 -qRT-Fwd	CTGGCAGAGGAAGCTGGA
mbd5 -qRT-Rev	TCGGTCAGCAGGTAAGACTTG
mbd6 -qRT-Fwd	GAGGTGAGGGTGTATCTGCG
mbd6 -qRT-Rev	ACCAGGAGCTTGGACCATTG
cybrd1 -qRT-Fwd	TCTTCACAGCTGGGTTGGT
cybrd1 -qRT-Rev	CAGCGATCCCCAACACTATC
hephl1a -qRT-Fwd	ATGTTTGGCAGCCCAGAT
hephl1a -qRT-Rev	GGCAGATTTCCATACATGAACC
hephl1b -qRT-Fwd	TCTGGACTTTCAGGAGAGCAA
hephl1b -qRT-Rev	GGCCAGGTAGGTTCCCATAC
aco1 -qRT-Fwd	ACCATGATTGATGGGCTAGG
aco1 -qRT-Rev	CGCTTCTGCCTCAATACCTC
irp2 -qRT-Fwd	CATGTTCACCGAGCTGAGG
irp2 -qRT-Rev	GCAGACTCTAAATTATTCCAGAACG
nlgn1 -qRT-Fwd	CGTGCCAACGGAGGATGTAA
nlgn1 -qRT-Rev	TTCACGAATGTCTCCTTTTCTACA
nlgn4xb -qRT-Fwd	TCACTGATATGCTGCCCGTC
nlgn4xb -qRT-Rev	ATATGAGCCGCCGTGAATGT
gabbr2 -qRT-Fwd	GTGAAGGTGGGCGAGTACAA
gabbr2 -qRT-Rev	ACGCACAAATGTCCGATCCT
gria2b -qRT-Fwd	GCCAAGGACTCTGGAAGTAAGG
gria2b -qRT-Rev	TCTGTGCATTCTTTGCCACCT
grin2db -qRT-Fwd	TTGTGTACAAGCGGGCAGAT
grin2db -qRT-Rev	CTCCAGAAAAGCCGAGGGAG
shank1 -qRT-Fwd	TATCGCTCTGCGCTCCAAAT
shank1 -qRT-Rev	GGGTGCTGGTAGTGACAGTG
dlgap4b -qRT-Fwd	GCATGTCACGAAGGCGTTTC
dlgap4b -qRT-Rev	CATGTGGAGACTGCTCTCGC
cntnap1 -qRT-Fwd	CCTATCAGGCGCCTTGTCAT
cntnap1 -qRT-Rev	TAGTGCCAGGCTTGAAGGTG
ptchd1 -qRT-Fwd	TGGCACTAGGATCAACGACG
ptchd1 -qRT-Rev	ACGCAGCTGGATCACTGAAA
rab3ab -qRT-Fwd	TCGGCATCGACTTCAAGGTG
rab3ab -qRT-Fwd	CCACGGTAATAAGCGGTGGT
slc6a13 -qRT-Fwd	ACCTTGACAACGCCACATCA
slc6a13 -qRT-Fwd	ATGTCCTCGATCCCAGACGA
kcnq3 -qRT-Fwd	AACCTCATTTACGACGCGCT
kcnq3 -qRT-Rev	GTGAGCCGAATCCTTCTCGT
β -actin-qRT-Fwd	ACGAACGACCAACCTAAACTCT
β -actin-qRT-Rev	TTAGACAACTACCTCCCTTTGC

Table S4. qRT-PCR primers for validating CLIP-seq candidates

id	gene	refseq	Fwd primer	Rev primer
z1	hbae3	NM_183066.3	CCACAGCTAACCAAGCAACC	GCTTTGGGTGCAACCTTGTC
z2	hbae3	NM_183066.3	CCCACAGCTAACCAAGCAAC	CCTGGAAAGAGTCTCACGGC
z3	hbae3	NM_183066.3	GGTGAAGAAGCACGGAACCA	TCCACGGAAACATGCACCTC
z4	hbae3	NM_183066.3	TCCTGAGGTGCATGTTTCCG	GCGGTACTTCTCGGACAGG
z5	gabbr2	NM_001144043.1	ATCCCAGCACTGAACGACAG	CCATGACCCTGTAGGAGGGA
z6	gabbr2	NM_001144043.1	CCAATGAGCTGGAGAAGGCA	TTGTACTCGCCCACCTTCAC
z7	gabbr2	NM_001144043.1	GTGAAGGTGGGCGAGTACAA	ACGCACAAATGTCCGATCCT
z8	gabbr2	NM_001144043.1	GGAGACGAGAAACGTGAGCA	TAGCAGCATCCGGGTTTGTT
z9	gabbr2	NM_001144043.1	CGGGATGCCTACTCTTGTCC	ATCCACTGGTACTTGCTGCC
z10	gabbr2	NM_001144043.1	GGGAACGCATGGGAACCATA	GCGGTTCGACACCTTGAAAC
z11	gabbr2	NM_001144043.1	GGCAAATTAACGTCCCGCTC	GATTGCGATGTCTCGTCCCT
z12	grin2bb	NM_001128337.2	TCATCTACGATGCAGCGGTC	TTTGGCAGTACGCAACAAGC
z13	grin2bb	NM_001128337.2	CCTGCAAGCGATGTGATTCG	TTCACCCCCACGGAATAAGC
z14	grin2bb	NM_001128337.2	TCCCCATTCTGGGTATCCGT	ATCCCCTGCCACCAAAGATG
z15	grin2bb	NM_001128337.2	AGTCTGGCCTGCCTGATCTA	AAACTAGTGCATGCTCCCCC
z16	grin2bb	NM_001128337.2	TACAGTCCCCTGCCGAAAAC	ACCAAACCCCAAAGAAGCCA
z17	grin2bb	NM_001128337.2	CATCTTTGGTGGCAGGGGAT	GGAGAGGTTGCGTCCTTCAA
z18	grin2bb	NM_001128337.2	TGCCTCTTCCACCGAAAGTC	ATCATCCCCGAACACCACAC
z19	grin2bb	NM_001128337.2	AGACAATGGGAGTTGGGCTG	AGGATCACCGCGATGCTAAG
z20	grin2bb	NM_001128337.2	GCTTATTCCGTGGGGGTGAA	TGCTCGAGCCATTAAACGGT
z21	nlgn1	NM_001142265.1	TGTAGAAAAGGAGACATTCGTGA	GGCAGTGATGGTAGAAGGCA
z22	nlgn1	NM_001142265.1	CGTGCCAACGGAGGATGTAA	TTCACGAATGTCTCCTTTTCTACA
z23	nlgn1	NM_001142265.1	AGCCTGGATCTGCCATTCTT	CTTTCACGAATGTCTCCTTTTCT
z24	Actb		ACGAACGACCAACCTAAACTCT	TTAGACAACTACCTCCCTTTGC

Table S5. PR-DUB and PRC1 components detected in zebrafish Mbd5 co-IP and mass spec

MBD5-interacting complexes from co-IP and MS	Accession	Protein	# detected in 3 Rounds of MS
PR-DUB components	F1Q5H5	Asxl1	3 rounds
	F1Q750	Hcfc1a	2 rounds
	A0A0R4IP04	Ogt.1	1 round
	A1L2G3	Bap1	1 round
	F6NIA2	Kdm1a	1 round
Non-canonical PRC1 variants	F1R653	Hdac1	3 rounds
	F1QNF2	CBX3a	1 round

Table S6. Proteins detected in three rounds of zebrafish Mbd5 co-IP and mass spec

Acession	Gene Symbol	1st Coverage(%)	2nd Coverage(%)	3rd Coverage(%)	Average Coverage(%)
A2VCY3	mbd5	43	39	48	43.33333333
F8W493	ywhabb	38	42	48	42.66666667
Q6NWH7	ddx5	28	25	22	25
Q7SXL4	nme2b.2	11	11	30	17.33333333
Q32PT3	hmgb2a	13	19	19	17
Q6PBI5	rps25	7	21	23	17
A0A0R4IDY9	hdac1	20	8	16	14.66666667
B2GTJ8	ywhaz	11	13	14	12.66666667
E9QBU9	eif2s3	19	4	8	10.33333333
Q6IQH4	psmd8	9	4	16	9.666666667
A0A2R8RNP8	xpo1b	17	4	3	8
Q804G7	anxa4	3	8	12	7.666666667
A0A2R8QE49	gna15.3	4	8	8	6.666666667
Q6P6X8	asxl1	14	2	3	6.333333333
Q6PFS4	immt	11	3	5	6.333333333
F6NK19	ap2b1	9	4	5	6
A0A0H2UKW6	akap8l	10	1	5	5.333333333
B8JLZ3	anxa1b	3	5	8	5.333333333
F1R9Y9	etfb	7	4	4	5
Q6PUS3	cstf3	8	5	1	4.666666667
Q6IQR1	adsl	4	2	5	3.666666667
Q66HW3	pip5k1bb	3	5	3	3.666666667
A0A2R8RQC2	fubp1	4	1	5	3.333333333
Q6TGS6	yars	5	2	2	3
A0A0R4IQN8	naca	6	1	1	2.666666667
A0A2R8QAT8	tars	3	1	3	2.333333333
A5A5E1	cyfip2	3	1	3	2.333333333
